# Metabolic Roles of HIF1, c-Myc, and p53 in Glioma Cells

**DOI:** 10.3390/metabo14050249

**Published:** 2024-04-25

**Authors:** Cristina Trejo-Solís, Rosa Angélica Castillo-Rodríguez, Norma Serrano-García, Daniela Silva-Adaya, Salvador Vargas-Cruz, Elda Georgina Chávez-Cortéz, Juan Carlos Gallardo-Pérez, Sergio Zavala-Vega, Arturo Cruz-Salgado, Roxana Magaña-Maldonado

**Affiliations:** 1 Laboratorio Experimental de Enfermedades Neurodegenerativas, Departamento de Neurofisiología, Laboratorio Clínico y Banco de Sangre y Laboratorio de Reprogramación Celular, Instituto Nacional de Neurología y Neurocirugía, Ciudad de Mexico 14269, Mexico; norma.serrano@innn.edu.mx (N.S.-G.); danieladaya@ciencias.unam.mx (D.S.-A.); sergio.zavala@innn.edu.mx (S.Z.-V.); 2 CICATA Unidad Morelos, Instituto Politécnico Nacional, Boulevard de la Tecnología, 1036 Z-1, P 2/2, Atlacholoaya 62790, Mexico; racastillo@ipn.mx; 3 Centro de Investigación Sobre el Envejecimiento, Centro de Investigación y de Estudios Avanzados (CIE-CINVESTAV), Ciudad de Mexico 14330, Mexico; 4 Departamento de Cirugía, Hospital Ángeles del Pedregal, Camino a Sta. Teresa, Ciudad de Mexico 10700, Mexico; darinka.velazquez@saludangeles.com; 5 Facultad de Odontología, Universidad Autónoma de Yucatán, Merida 97000, Mexico; elda.chavez@correo.uady.mx; 6 Departamento de Fisiopatología Cardio-Renal, Departamento de Bioquímica, Instituto Nacional de Cardiología, Ciudad de Mexico 14080, Mexico; jcgallardo@ciencias.unam.mx; 7 Centro de Investigación Sobre Enfermedades Infecciosas, Instituto Nacional de Salud Pública, Cuernavaca 62100, Mexico; investigador70@insp.mx

**Keywords:** metabolism, AKT, HIF1, c-Myc, p53, glioma, therapeutic targets

## Abstract

The metabolic reprogramming that promotes tumorigenesis in glioblastoma is induced by dynamic alterations in the hypoxic tumor microenvironment, as well as in transcriptional and signaling networks, which result in changes in global genetic expression. The signaling pathways PI3K/AKT/mTOR and RAS/RAF/MEK/ERK stimulate cell metabolism, either directly or indirectly, by modulating the transcriptional factors p53, HIF1, and c-Myc. The overexpression of HIF1 and c-Myc, master regulators of cellular metabolism, is a key contributor to the synthesis of bioenergetic molecules that mediate glioma cell transformation, proliferation, survival, migration, and invasion by modifying the transcription levels of key gene groups involved in metabolism. Meanwhile, the tumor-suppressing protein p53, which negatively regulates HIF1 and c-Myc, is often lost in glioblastoma. Alterations in this triad of transcriptional factors induce a metabolic shift in glioma cells that allows them to adapt and survive changes such as mutations, hypoxia, acidosis, the presence of reactive oxygen species, and nutrient deprivation, by modulating the activity and expression of signaling molecules, enzymes, metabolites, transporters, and regulators involved in glycolysis and glutamine metabolism, the pentose phosphate cycle, the tricarboxylic acid cycle, and oxidative phosphorylation, as well as the synthesis and degradation of fatty acids and nucleic acids. This review summarizes our current knowledge on the role of HIF1, c-Myc, and p53 in the genic regulatory network for metabolism in glioma cells, as well as potential therapeutic inhibitors of these factors.

## 1. Introduction

Gliomas are the most common tumors of the primary central nervous system (CNS), with Glioblastoma (GBM) being the most malignant primary tumor [1]. Despite ongoing efforts to improve standard diagnosis and treatment, the median survival of patients with GBM is 14.6 months [2]. At the cellular level, GBM is characterized by increased cell proliferation, angiogenesis, necrotic and hypoxic areas, and metabolic alterations leading to tumor heterogeneity and the capacity to migrate to healthy brain parenchyma [3]. The latest WHO classification of CNS tumors, which includes histological and molecular features, regards GBM tumors as carrying wild-type isocitrate dehydrogenase (IDHwt) variants, even those not showing microvascular proliferation or necrosis. At a molecular level, GBM shows at least one of the following features: an epidermal growth factor receptor, telomerase reverse transcriptase promoter mutation, or chromosome 7 gain/10 loss [4]. Astrocytoma with mutated isocitrate dehydrogenase (IDH) is classified in various malignancy grades, determined by the lack of α-thalassemia mental retardation X-linked (ATRX) and cyclin-dependent kinase inhibitor (CDKN)2A/B status [5].

Additionally, GBM has been described to exhibit mutations, translocations, deletions, and amplifications involved in the downregulation of phosphatase and tensin homolog (PTEN) [6], F-box, WD40 domain protein 7 (FBXW7) [7], neurofibromatosis1 (NF1), p14 alternative reading frame (ARF), tuberous sclerosis proteins ½ (TSC1/2), Von Hippel–Lindau protein (pVHL) [8], liver kinase B (LKB1) [9], and anti-apoptotic proteins such as Bax, Bad, and the apoptotic protease activating factor-1 (Apaf-1), caspase-8, and caspase-7 [10,11,12,13]. However, other genetic alterations induce the overexpression of anti-apoptotic proteins, including B cell lymphoma 2 (Bcl-2), B cell lymphoma-extra-large (Bcl-xL), mutated IDH1, pyruvate kinase M2 (PKM2) [14], Ras homologue enriched in brain (Rheb), eukaryotic translation initiation factor 4E (eLF-4E), murine double minute 2 (mdm) [15,16], and growth factor receptors (epidermal growth factor receptor (EGFR), platelet-derived growth factors receptors (PDGFRs), and the vascular endothelial growth factor receptor (VEGFR)). These genic alterations have been described to modulate the activation of several signaling pathways, including phosphoinositide-3-kinases (PI3K)/protein kinase B (AKT) and rat sarcoma virus (RAS)/rapidly accelerated fibrosarcoma (RAF)/MEK/extracellular signal-regulated kinase (ERK) [17,18,19,20,21].

The oncogenic activation of PI3K/AKT and RAS/RAF/MEK/ERK signaling pathway in glioblastoma either directly or indirectly promotes metabolic reprogramming. Directly, AKT and ERK promote an anabolic metabolism through phosphorylated key metabolic enzymes [22]. Moreover, AKT indirectly modulates various transcriptional factors such as forkhead box class O (FOXO), p53, sterol regulatory element-binding protein (SREBPS), hypoxia-inducible factor-1 (HIF1), and cellular-Myc (c-Myc), which are master regulators of cellular metabolism via downstream effects such as mammalian target of rapamycin complex 1 (mTOR), glycogen synthase kinase 3β (GSK3β), and mdm2 [23]. The over-activation of HIF1 and c-Myc regulates the expression of key gene groups involved in metabolism to support cellular proliferation angiogenesis, migration, chemo- and radio-resistance, and cell death resistance in glioma cells [24,25,26]. Meanwhile, the tumor-suppressing protein p53, often lost in glioblastoma, negatively regulates HIF1 and c-Myc and modulates the balance among the glycolysis and oxidative phosphorylation (OXPHOS). These alterations in this triad of transcriptional factors induce a metabolic shift in glioma cells that allows them to adapt and survive drastic changes in the tumor microenvironment [22,23].

**Figure 1:**
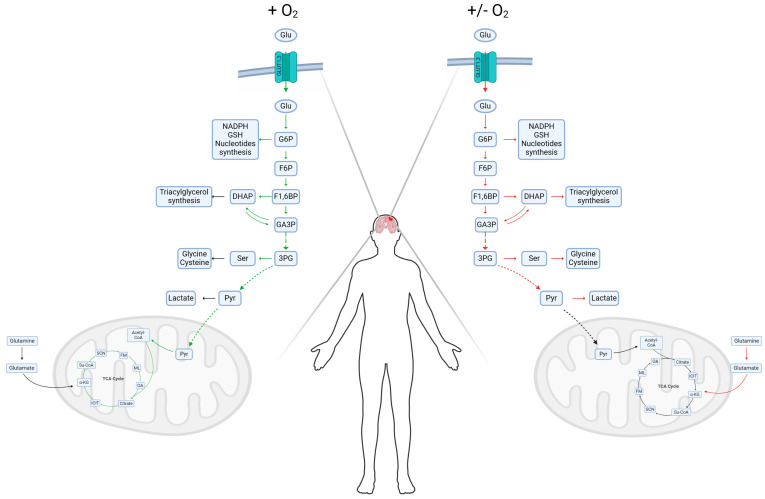
Metabolic differences between normal and cancer cells. Glucose metabolism in normal cells produces pyruvate, converted to acetyl-CoA and incorporated into the TCA cycle, followed by OXPHOS. In glioma cells, glucose is the primary energy source metabolized through the glycolytic pathway (Warburg effect). Under reduced glucose levels, GBM cells use glutamine to produce αKG. Abbreviations: dihydroxyacetone phosphate (DHAP), fructose-6-phosphate (F6P), fructose-1,6 biphosphate (F1,6BP), fumarate (FM), glucose (Glu), glucose-6-phosphate (G6P), glucose transporters 1,3 (GLUT1,3), gluthatione (GSH), glyceraldehyde-3-phosphate (GA3P), isocitrate (ICIT), α-ketoglutarate (α-KG), malate (ML), nicotinamide adenine dinucleotide phosphate (NADPH), oxalacetate (OA), oxidative phosphorylation (OXPHOS), 3-phosphoglycerate (3PG), pyruvate (Pyr), serine (Ser), succinate (SCN), succinyl-coenzyme A (Su-CoA). ↑ activation. The figure was created with BioRender.com.

GBM cells mainly obtain energy adenosine triphosphate (ATP) and precursors to synthesize nucleic acids, carbohydrates, lipids, and proteins through the glycolytic pathway (Warburg effect). However, they also utilize other energy sources such as glutamine, fatty acids, lactate, and acetate, which depend on genetic alterations and the tumor microenvironment [27,28,29,30]. Glucose (Glu) is the main energy source in GBM cells regardless of their carcinogenic origin rather than OXPHOS because the glycolytic process generates ATPs faster than OXPHOS and metabolic intermediates [31,32,33]. Glycolysis generates two molecules of pyruvate, which is converted mainly to lactate, 2NADH, 2ATP, and metabolic intermediates such as glucose 6-phosphate (G6P), dihydroxyacetone phosphate (DHAP), and 3-phosphoglycerate (3PG) for the synthesis of nucleotides, NADPH, glutathione (GSH), and lipids, as well as amino acids, respectively. However, under low-glucose conditions, GBM cells primarily utilize glutamine to produce α-ketoglutarate (α-KG), which is incorporated into the Krebs cycle for anabolic precursors such as citrate, malate, ATP, NADH, and FADH2, where the latter is directed to OXPHOS to produce high concentrations of energy (34 ATP) [33,34]. However, normal mammalian cells obtain energy for survival and growth through glucose and OXPHOS. Pyruvate obtained during the glycolytic pathway is converted to acetyl-CoA, and subsequently, it is incorporated into the tricarboylic acid cycle (TCA cycle), followed by OXPHOS [35]. Understanding the mechanisms that induce metabolic differences between normal and cancer cells would help us to develop more efficient and specific therapeutic strategies against cancer cells (Figure 1).

This review describes the cellular functions of PI3K/AKT/mTOR and RAS/RAF/MEK/ERK signaling pathways due to their critical role in the regulation of the metabolism as well as the role of the transcriptional factors c-Myc, HIF1, and p53 in modulating the pathways involved in energy metabolism like glycolysis, pentose phosphate cycle, TCA cycle, OXPHOS, fatty acid (FA) synthesis and degradation, the mevalonate route, and the metabolism of amino acids and nucleic acids in healthy and glioma malignant cells. Potential therapeutic molecules that modulate these transcriptional factors are described in this study.

## 2. Regulation of Cell Metabolism by the PI3K/AKT Pathway in GBM

**Figure 2:**
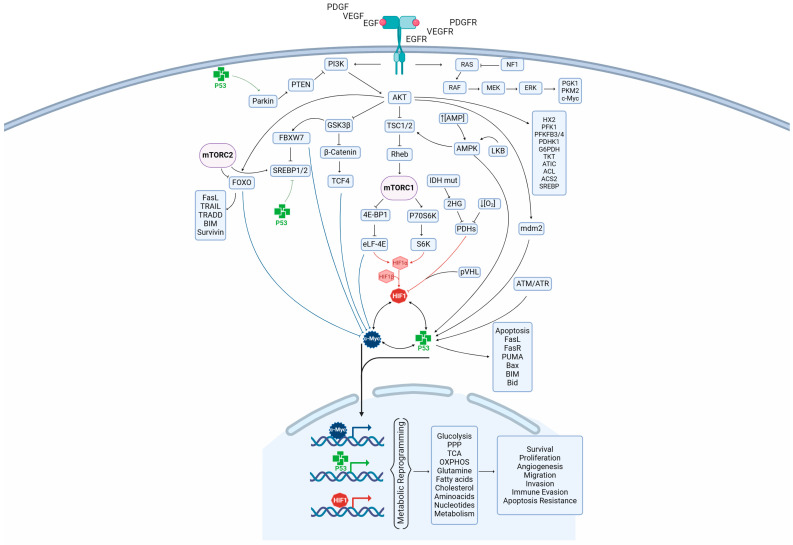
Metabolic reprogramming modulates transcriptional factors p53, c-Myc, and HIF1. PI3K/AKT and RAS/Raf/MEK/ERK Signaling pathways contribute to glioma malignancy. The RAS/Raf/MEK/ERK pathway activates glycolysis via PGK1, PKM2, and c-Myc. EGF/EGFR/PI3K/AKT signaling and their downstream effectors, such as mTORC1, c-Myc, HIF1, mdm2, p53, FOXO, and SREBP, regulate metabolic processes such as glycolysis, tricarboxylic acid cycle, pentose phosphate pathway, fatty acid synthesis and degradation, and mevalonate pathway. Abbreviations: ATP-citrate lyase (ACL), acyl-CoA synthetase 2 (ACS2), protein kinase B (AKT), adenosine-5′-monophosphate (AMP), AMP-activated protein kinase (AMPK), 5-aminoimidazole-4-Carboxamide Ribonucleotide Formyltransferase/IMP Cyclohydrolase (ATIC), ataxia-telangiectasia mutated (ATM), adenosine triphosphate (ATP), ataxia telangiectasia and Rad3 related (ATR), BH3 interacting-domain death agonist (Bid), Bcl-2 Interacting Mediator of cell death (BIM), F-box and WD40 domain protein 7 (FBXW7), epidermal growth factor (EGF), epidermal growth factor receptor (EGFR), eukaryotic translation initiation factor 4E-binding protein 1 (4E-BP1), eukaryotic translation initiation factor 4E (eIF-4E), extracellular signal-regulated kinase (ERK), Fas ligand (FasL), forkhead box class O (FOXO), glucose-6-phosphate dehydrogenase (G6PDH), glycogen synthase kinase 3β (GSK3β), hexokinase 2 (HX2), hypoxia-inducible factor 1 (HIF1), 2-hydroxiglutarate (2HG), isocitrate dehydrogenase mutants (IDH mut), liver kinase B (LKB), murine double minute 2 (mdm2), mammalian target of rapamycin complex 1 (mTORC1), neurofibromatosis 1 (NF1), platelet-derived growth factor receptors (PDGFR), phosphoglycerate kinase 1 (PGK1), phosphofructokinase1 (PFK1), phosphofructo-2-kinase/fructose-2,6-biphosphatase 3/4 (PFKFB3/4), pyruvate dehydrogenase kinase1 (PDHK1), phosphoinositide-3-kinases (PI3K), pyruvate kinase isozyme type M2 (PKM2), pentose phosphate pathway (PPP), phosphatase and tensin homolog (PTEN), p53 upregulated modulator of apoptosis (PUMA), rapidly accelerated fibrosarcoma (RAF), rat sarcoma virus (RAS), Ras homologue enriched in brain (RheB), ribosomal protein S6 kinase (S6K), sterol regulatory element-binding protein (SREBP), transcription factor 4 (TCF-4), transketolase (TKT), TNFR1-associated death domain protein (TRADD), tumor necrosis factor-related apoptosis-inducing ligand (TRAIL), tuberous sclerosis proteins 1/2 (TSC1/2), vascular endothelial growth factor receptor (VEGFR), and Von Hippel–Lindau protein (pVHL).↑ activation, ⊥ inhibition. The figure was created with BioRender.com.

A strong relationship has been suggested between oncogenic signaling pathways and metabolic reprogramming towards a more glycolytic phenotype independent of oxygen availability (Warburg effect), which satisfies the energetic needs to synthesize carbohydrates, amino acids, lipids, and nucleic acid that favor the reproduction and dissemination of glioma cells. In this sense, the signaling pathway EGFRvII/PI3K/AKT promotes a glycolytic phenotype (Figure 2) in glioma cells by increasing the expression and membrane translocation of glucose transporters 1 and 3 (GLUT1 and GLUT3) [36,37,38] and the phosphorylation and activation of glycolytic enzymes such as hexokinase2 (HX2) [39,40], phosphofructokinase1 (PFK1) [41], phosphofructo-2-kinase/fructose-2,6-biphosphatase (PFKFB4,-3) [42,43], and pyruvate dehydrogenase kinase 1 (PDHK1) [44].

The PI3K/AKT pathway could induce the upregulation of the pentose phosphate pathway (PPP), promoting the stability of glucose-6-phosphate dehydrogenase (G6PDH), the key enzyme of this pathway, by inhibiting the E3 ligase tripartite motif containing 21 (E3-TRIM21) of G6PDH [45]. Furthermore, AKT increases the nonoxidative phase of PPP by phosphorylating transketolase (TKT) at Thr382 and promoting purine synthesis through ribose 5-phosphate (R5P) [46]. R5P is then converted into phosphoribosylpyrophosphate (PRPP) by PRPP synthetase [46,47].

Then, PI3K/AKT signaling regulates purine de novo and salvage synthesis by modulating PRPP levels as well as 5-aminoimidazole-4-Carboxamide Ribonucleotide Formyltransferase/IMP Cyclohydrolase (ATIC) activity [47]. In addition, Ben-Sahra et al. demonstrated that the AKT/mTOR/ribosomal protein S6 kinase (S6K) pathway activates the enzyme catalyzing the first step of pyrimidine synthesis by phosphorylating carbamoyl-phosphate synthetase 2, aspartate transcarbamoylase, and dihydroorotase (CAD) at Ser1859 [48]. 

PI3K/AKT signaling also upregulates the synthesis of FA by directly phosphorylating ATP-citrate lyase (ACL). ACL can regenerate acetyl-CoA cytosolic levels by converting citrate into oxalacetate (OA) and acetyl-CoA, whereas acetyl-CoA can synthesize mevalonate and FA [43]. AKT also promotes the synthesis of cholesterol and FA by phosphorylating GSK3β, which activates FBXW7, a ubiquitin ligase of SREBP transcriptional factor [49] that promotes the transcription of target genes such asACL, fatty acid synthase (FAS), acetyl-CoA carboxylase (ACC), and steaoryl-CoA-desaturase-1 (SCD 1) to promote the synthesis of FA and the expression of low-density lipoprotein receptors to increase cholesterol uptake by malignant cells [50].

On the other hand, PI3K/AKT regulates cellular metabolism by modulating transcriptional factors. AKT regulates the activation of the tumor-suppressing protein p53 by phosphorylating mdm2 ubiquitin ligase at Ser166,186, leading to the nuclear translocation of mdm2, which negatively induces p53 degradation [51]. However, under metabolic stress conditions, such as energy and nutrient deprivation, the pathway promotes the stability of p53 by phosphorylating it at Ser15 via the activation of AMP-activated protein kinase (AMPK) and the inhibition of AKT [52], whilst AMPK is phosphorylated by LKB kinase to activate the protein TSC2 [53].

Furthermore, AKT activates glycolysis and suppresses apoptosis by phosphorylating FOXOs in the nucleus, which in turn activates c-Myc [54]. FOXO inhibits c-Myc by increasing the levels of microRNA (miR)-34c and miR-145, which destabilizes c-Myc mRNA and suppresses its transcription [55,56]. On the other hand, the mTORC2 can inhibit FOXOs by acetylating FOXO1 and FOXO3, promoting c-Myc activation and enhancing the Warburg effect in glioblastoma cells [54]. 

Additionally, c-Myc induces indirect p53 activation by increasing the levels of p19ARF, which binds and inhibits mdm2 [57]. c-Myc inhibits BIM1, which in turn inhibits p19ARF, promoting p53 stability [58]. However, p19ARF is directly inhibited at c-Myc by the independent mechanism of p53 [59]. In addition, c-Myc also promotes p53 activity via mirRNA-34a. mirRNA-34a blocks Sirtuin1 (SIRT1), which inhibits p53 [58]. However, AKT also induces the expression of c-Myc by inhibiting p53. p53 decreases c-Myc mRNA levels by inducing miR-145 [60]. Interestingly, p53 has also been shown to increase the levels of c-Myc [61]. 

PI3K/AKT signaling promotes the translation of HIF1α, c-Myc, and the SREBP mRNA via mTORC1. AKT inhibits TSC2 by phosphorylating it, which allows it to form a complex with TSC1. The TSC1/TSC2 complex mediates the inhibition of RHEB, an activator of mTOR. mTOR phosphorylates p70S6K, eIF-4E, and the eukaryotic translation initiation factor 4E binding protein (eIF-4EBP1). Phosphorylation of eIF-4EBP1 results in the suppression of its inhibitory bind to eIF-4E, whereas p70S6K is activated by phosphorylation of the ribosomal protein S6, promoting the synthesis of HIF1α and c-Myc [21,62]. Meanwhile, mTORC1 induces the transcription of SREBP-1c, independently of the activity of ribosomal protein S6. On the other hand, the pathway RAS/RAF/MEK/ERK induces glycolysis by activating phosphoglycerate kinase 1 (PGK1) [63] and PKM2 [64] (Figure 2).

## 3. Role of p53 in Cell Metabolism

The tumor-suppressing protein p53 is activated by several stimuli, including DNA damage, ribosomal and oxidative stress, starvation, hypoxia/anoxia, oncogene activation, and anticancer drugs [65]. Under normal conditions, p53 levels remain low due to its continuous ubiquitination and degradation by mdm2 [66]. mdm4 (mdmx), a homolog protein to mdm2 [67], can inactivate p53 either by binding it directly, inhibiting the transactivation ability of p53, or by binding mdm2 to form a p53-mdm2-mdmx complex, which promotes the ubiquitin ligase activity of mdm2 [68]. On the other hand, the interaction between p53 and mdm2 is disrupted by the suppressor tumor protein (p19ARF) binding to mdm2, inhibiting its E3 ligase activity and favoring p53 stability [69]. The p19ARF/mdm2/p53 pathway does not function only as a simple linear pathway if not as a complex signaling network [70]. mdm2 and p19ARF can function via p53-independent mechanisms [70]. p19ARF binds to numerous target proteins such as Cyclin D, E2F, and transcriptional factors c-Myc, HIF1α, and BCL6 [71,72,73] and induces posttranslational regulation such as sumoylation (mdm2) or protein turnover of C-terminal-binding proteins (CTBP1/2) proteins, which can promote cellular senescence, cell cycle arrest, and apoptosis [70,71,74,75]. Understanding the interactions off and on the p19ARF/mdm2/p53 pathway would allow us to more efficiently develop therapeutic strategies against cancer [70]. Furthermore, p53 accumulation in response to DNA damage is further promoted after its phosphorylation by ataxia-telangiectasia mutated (ATM) and ataxia telangiectasia and Rad3 related (ATR) kinases, hindering its interaction with mdm2 [76]

**Figure 3:**
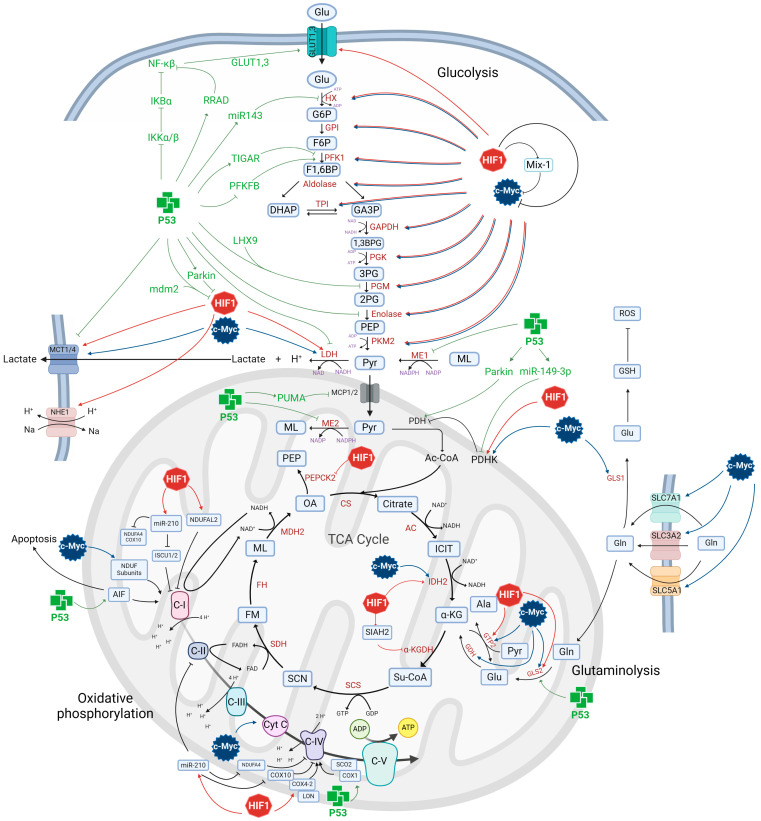
Role of transcriptional factors p53, c-Myc, and HIF1 in the regulation of glycolysis, oxidative phosphorylation, TCA cycle, and glutaminolysis. p53 inhibits the translocation of GLUT1,3 by inhibiting nuclear NF-κB. It also inhibits HX, PGM, enolase, PFKFB, MCT1/4, ME1, PDHK, and MCP 1/2 but activates PDH, GLS2, and complexes I and IV of the ETC. c-Myc and HIF1 positively regulates the transcription of most glycolytic pathway enzymes and LDH, MCT1/4, PDHK, IDH2, GTP2, GLS2, and complex I of the ETC. Moreover, c-Myc positively regulates the transcription of SLC7A1, SLC3A2, SLC5A1, GDH, GLS1, and HIF1 and induces the transcription of NHE1, PEPCK2, and complex IV of the ETC. Abbreviations: aconitase (AC), alanine (Ala), adenosine diphosphate (ADP), adenosine triphosphate (ATP), apoptosis-inducing factor (AIF), 1,3-bisphosphoglycerate (1,3BPG), cyclooxygenase 2 (COX-2), citrate synthase (CS), cytochrome c (Cyt C), cytochrome c oxidase (SCO2), dihydroxyacetone phosphate (DHAP), electron transport chain (ETC), flavine adenine dinucleotide (FAD), fructose-6-phosphate (F6P), fructose-1,6 biphosphate (F1,6BP), fumarate (FM), glucose (Glu), glucose-6-phosphate (G6P), glucose transporters 1,3 (GLUT1,3), glutamate dehydrogenase (GDH), glutamine (Gln), gluthatione (GSH), guanosine diphosphate (GDP), guanosine triphosphate (GTP), glyceraldehyde-3-phosphate (GA3P), glutaminase1,-2 (GLS1,2), glutamic-pyruvic transaminase 2 (GTP2), hexokinase (HX), hypoxia-inducible factor-1 (HIF1), IkappaB kinase (IKK), isocitrate (ICIT), isocitrate dehydrogenase (IDH2), α-ketoglutarate (α-KG), α-ketoglutarate dehydrogenase (α-KGDH), lactate dehydrogenase (LDH), LIM Homeobox Protein 9 (LHX9), malate (ML), malate dehydrogenase (MDH2), malic enzyme 1 (ME1), monocarboxylate transporter 1/4 (MCT1/4), mitochondrial pyruvate carrier (MCP 1/2), murine doble minute 2 (mdm2), Na+/H+ exchanger (NHE1), NADH:ubiquinone oxidoreductase (NDUF), dehydrogenase [ubiquinone] 1 alpha subcomplex and 4-like 2 (NDUFA4L2), nicotinamide adenine dinucleotide phosphate (NADPH), nuclear factor kappa-light-chain-enhancer of activated B cells (NF-κB), oxalacetate (OA), phosphoenolpyruvate (PEP), phosphoenolpyruvate carboxykinase 2 (PEPCK2), 6-phosphofructo-2-kinase/fructose-2,6-bisphosphatase (PFKFB), 2-phosphoglycerate (2PG), 3-phosphoglycerate (3PG), phosphoglycerate mutase (PGM), p53 upregulated modulator of apoptosis (PUMA), pyruvate dehydrogenase (PDH), pyruvate dehydrogenase kinase (PDHK), Ras-related associated with diabetes (RRAD), reactive oxygen species (ROS), succinate (SCN), succinyl-coenzyme A (Su-CoA), succinyl- CoA synthetase (SCS), succinate dehydrogenase (SDH), solute carrier family 5 member 1 (SLC5A1), solute carrier family 7 member 1 (SLC7A1), solute carrier family 3 member 2 (SLC3A2), TP53-induced glycolysis and apoptosis regulator (TIGAR), and tricarboxylic acid cycle (TCA). ↑ activation, ⊥ inhibition. The figure was created with BioRender.com.

p53 is a transcriptional factor that regulates the genic expression of proteins that modulate apoptosis, autophagy, the cell cycle, DNA repair, senescence, and metabolism to prevent tumorigenesis [77,78]. In most cell types, p53 inhibits glycolysis and favors OXPHOS through several mechanisms [79] (Figure 3). p53 reduces glucose (Glu) uptake by directly repressing the transcription of the Glu transporters GLUT1 and GLUT4 [80]. Liu et al. demonstrated that p53 induces the transcription of Ras-related protein associated with diabetes (RRAD), which binds to the p65/nuclear factor kappa-light-chain-enhancer of activated B cells (NF-κB) subunit, inhibiting the nuclear translocation and activity of NF-κB to restrict the localization to the plasma membrane and activity of GLUT1 and GLUT3 [81]. p53 inhibits the transcriptional activity of NF-κB by blocking the IkappaB kinases IKKα and IKKβ, which in turn promote the activation of IκBα, an inhibitor of NF-κB [82].

In addition, p53 blocks catalytic reactions of the glycolytic pathway. For example, p53 reduces the formation of glucose 6-phosphate (G6P) from Glu by increasing the expression of miR143, an inhibitor of HX2 mRNA [83]. p53 also suppresses the conversion of fructose 6-phosphate (F6P) into fructose-1,6-biphosphate (F1,6BP) by transcriptionally inducing the TP53-induced glycolysis and apoptosis regulator (TIGAR), which reduces the intracellular levels of fructose-2,6-bisphosphate (F2,6BP), an allosteric activator of PFK1 [84]. p53 reduces the intracellular levels of F2,6BP by transcriptionally repressing 6-phosphofructo-2-kinase/fructose-2,6-biphosphatase (PFKFB)-3 and -4 [85]. In addition, both the conversion of 3-phosphoglycerate (3PG) into 2-phosphoglycerate (2PG) and 2PG into phosphoenolpyruvate (PEP) is blocked by p53 by downregulating the levels of phosphoglycerate mutase 1 (PGAM1) and repressing the expression of enolase 3 mRNA, respectively [86,87]. Huang et al. demonstrated that the inhibition of p53 by statins increases the activity of enolase 3 and, consequently, lactate levels [87].

p53 also blocks lactate production and inhibits lactate transport into and out of tumor cells by negatively regulating lactate dehydrogenase A (LDHA) and reducing the stability of the monocarboxylate transporter 1 (MCT1) mRNA [88] or inducing the degradation of HIF1α, a transcriptional regulator of LDHA and MCT1 [89,90].

p53 indirectly regulates the expression of glycolytic enzymes by modulating signaling pathways such as mTOR and PI3K/AKT. p53 induces the expression of parkin, which positively regulates PTEN, leading to the negative regulation of PI3K/AKT [91]. p53 senses nutrient levels by inducing the expression of LKB2, increasing AMPK phosphorylation, and inducing the GTPase capacity of TSC2, thereby inhibiting mTOR [92]. mTOR inactivation induces downregulation in GLUT1, PFK, and PDHK1 mRNA, blocking HIF1α expression [93]. p53 also activates AMPK by increasing the phosphorylation of the AMPKα subunit [94]. Conversely, AMPK induces the phosphorylation of p53 at Ser15, promoting its stability [52]. 

**Figure 4:**
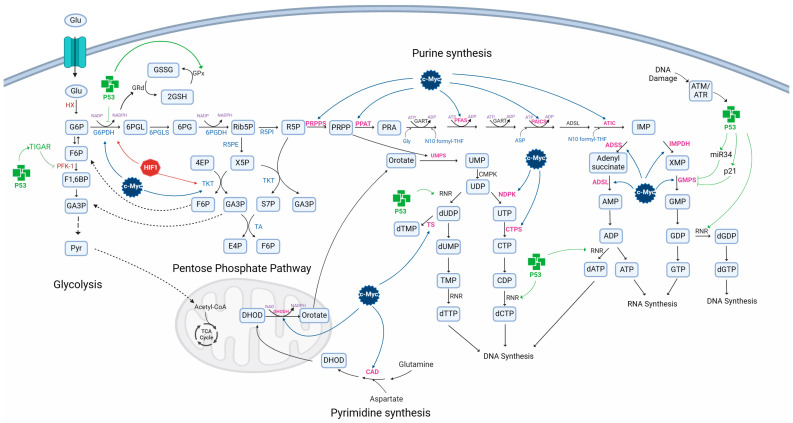
Involvement of transcriptional factors c-Myc, p53, and HIF1 in the regulation of glycolysis, pentose phosphate pathway (PPP), pyrimidine, and purine synthesis. p53 modulates PPP via PFK1 and G6PDH, and purine and pyrimidine synthesis through RNR and GMPS. C-Myc and HIF1 regulate PPP through transcription of G6PDH and TKT. In addition, c-Myc induces gene expression of PRPPS, PPAT, PFAS, PAICS, ATIC, ADSL, ADSS, IMPDH, NDPK, CTPS, CAD, DHOD, and TS. Abbreviations: acetyl- coenzyme A (Ac-CoA), adenylosuccinate lyase (ADSL), adenylosuccinate synthase (ADSS), adenosine diphosphate (ADP), adenosine-5′-monophosphate (AMP), adenosine triphosphate (ATP), aspartate (Asp), 5-aminoimidazole-4-Carboxamide Ribonucleotide Formyltransferase/IMP Cyclohydrolase (ATIC), ataxia-telangiectasia mutated (ATM), ataxia telangiectasia and Rad3 related (ATR), carbamoyl-phosphate synthetase 2, aspartate transcarbamoylase, dihydroorotase (CAD), cytidine biphosphate (CDP), cytidine triphosphate (CTP), cytidine triphosphate synthase (CTPS), cytidine/uridine monophosphate kinase (CMPK), deoxyadenosine triphosphate (dATP), deoxycytidine triphosphate (dCTP), deoxyguanosine triphosphate (dGTP), deoxythymidine monophosphate (dTMP), deoxythymidine triphosphate (dTTP), deoxyuridine monophosphate (dUMP), deoxyuridine diphosphate (dUDP), dihydroorotate dehydrogenase (DHOD), erythrose-4- phosphate (4EP), fructose-6-phosphate (F6P), fructose-1,6 biphosphate (F1,6BP), glicine (Gly), glucose (Glu), glucose-6-phosphate (G6P), glucose-6-phosphate dehydrogenase (G6PDH), glutathione (GSH), glutathione peroxidase (GPX), glutathione reductase (GRd), oxidized glutathione (GSSG), guanosine monophosphate synthetase (GMPS), guanosine diphosphate (GDP), guanosine triphosphate (GTP), guanosine monophosphate synthase (GMPS), glyceraldehyde-3-phosphate (GA3P), glycinamide ribonucleotide (GART), hexokinase (HX), inosine 5′-monophosphate dehydrogenase (IMPDH), inosine monophosphate (IMP), N-10-formyltetrahydrofolate (N10 formyl-THF), nicotinamide adenine dinucleotide (NAD), nicotinamide adenine dinucleotide phosphate (NADPH), nucleoside diphosphate kinase (NDPK), phosphofructokinase type 1 (PFK1), phosphoribosylformylglycinamide synthase (PFAS), 6-phosphogluconate (6PG), 6-phosphogluconolactona (6PGL), 6-phosphogluconolactonase (6PGLS), 6-phosphogluconate dehydrogenase (6PGDH), phosphoribosylaminoimidazole carboxylase (PAICS), phosphoribosylamine (PRA), phosphoribosylpyrophosphate amidotransferase (PPAT), phosphoribosylpyrophosphate (PRPP), phosphoribosylpyrophosphate synthetase (PRPPS), pyruvate (Pyr), ribonucleotide reductase (RNR), ribose- 5-phosphate (R5P), ribulose -5-phosphate (Rib5P), ribulose -5-phosphate epimerase (R5PE), ribulose -5-phosphate isomerase (R5PI), sedoheptulose- 6- phosphate (S6P), thymidylate synthase (TS), thymidylate monophosphate (TMP), uridine diphosphate (UDP), uridine monophosphate (UMP), uridine monophosphate synthetase (UMPS), xylose-5-phosphate (X5P), and xanthosine -5′-phosphate (XMP). ↑ activation, ⊥ inhibition. The figure was created with BioRender.com.

p53 also regulates PPP, directing the flux of G6P to the PPP via TIGAR [84] (Figure 4). However, it has been reported that p53 inhibits PPP by binding to monomers of G6PDH, thus hindering the formation of the active dimer and its enzymatic activity and decreasing the levels of GSH and NADPH, as well as pentoses, essential precursors for nucleotide and lipid biosynthesis [95].

p53 modulates TCA by reducing the mitochondrial enzyme PDHK2 levels and increasing the expression of miR-149-3p [83,96]. The inactivation of PDHK2 by p53 favors the catalytic activation of pyruvate dehydrogenase (PDH) [92]. However, p53 can also activate PDH via parkin [97,98]. PDH catalyzes the decarboxylation of pyruvate into acetyl-CoA, which enters Krebs’ cycle to produce FADH and NADH and up to 36 ATP moles per Glu mole in the presence of oxygen. p53 downregulates the levels of malic enzymes (ME)-1 and -2, which induce the formation of cytoplasmic and mitochondrial malate, as well as the production of NADPH from pyruvate, increasing intracellular pyruvate levels [99].

p53 stimulates OXPHOS by transcriptionally inducing cytochrome c oxidase 2 (SCO2) and cytochrome c oxidase 1 (COX1), two enzymes involved in the assembly of the COX IV complex of the electron transport chain (ETC) [95,100] (Figure 4). p53 upregulates the expression of apoptosis-inducing factor (AIF), which is required for the function of the mitochondrial complex I of the ETC [101] and for apoptosis induction by a caspase-independent mechanism [102]. In addition, p53 promotes glutaminse2 (GLS2) expression [103,104] (Figure 4), increasing the levels of glutamate and α-KG, which speed up the TCA cycle and OXPHOS [103,104]. p53 inhibits OXPHOS and favors glycolysis by inducing p53 upregulated modulator of apoptosis (Puma) transcription, which blocks the formation of the mitochondrial pyruvate carrier (MCP1/MCP2 complex), thereby inhibiting mitochondrial uptake of pyruvate in cancer cells [105].

**Figure 5:**
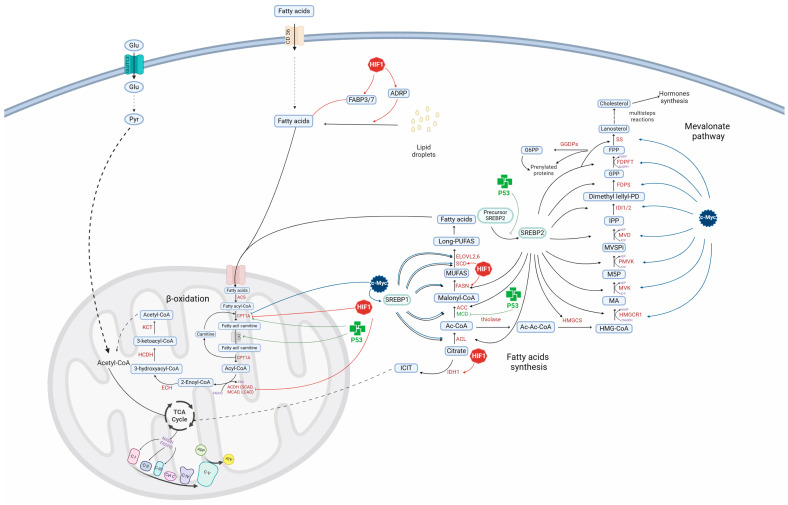
Participation of c-Myc, p53, and HIF transcriptional factors in regulating lipid metabolism, β- oxidation, fatty acid synthesis, and mevalonate pathway. p53 suppresses fatty lipid synthesis and cholesterol by inhibiting SREBP1,-2 but promotes β-oxidation. C-Myc positively regulates the mevalonate pathway and lipid synthesis via induction of HMGCR1, MVK, PMVK, MVD, IDI 1/2, FDPS, FDPFT, ACL, ACC, FASN, and SCD. c-Myc and HIF1 inhibit CPT1 transcription. HIF-1 induces ADRP, FABP 3/7, FASN, SCD, and IDH1, but inhibits MCAD and LCAD. Abbreviations: Acetyl-CoA carboxylase (ACC), acyl-CoA synthetase (ACS), ATP-citrate lyase (ACL), acetaldehyde dehydrogenase (ACDH), adenosine diphosphate (ADP), adipose differentiation-related proteins (ADRP), adenosine triphosphate (ATP), carnitine acyltransferases (CAT), carnitine palmitoyltransferase 1 (CPT1), coenzyme A (CoA), enoyl-CoA hydratase (ECH), fatty acids elongases (ELOVL), flavine adenine dinucleotide (FAD), fatty acid synthase (FAS), fatty acid binding proteins 3 and 7 (FABP 3/7), fatty acid synthase (FASN), farnesyl diphosphate synthase (FDPS), farnesyl diphosphate farnesyltransferase (FDPFT), farnesyl pyrophosphate (FPP), glucose (Glu), glucose transporters 1,3 (GLUT1,3), glucose-6-phosphate phosphatase (G6PP), geranylgeranyl diphosphate synthase (GGDPS), geranylgeranyl pyrophosphate (GPP), 3-hidroxiacil-CoA dehydrogenase (HACDH), hydroxy-3-methylglutaryl-coenzyme A (HMG-CoA), 3-hydroxy-3-methylglutaryl CoA reductase 1 (HMGCR1), hypoxia-inducible factor 1 (HIF1), isocitrate (ICIT), isocitrate dehydrogenase 1 (IDH1), isopentenyl diphosphate Δ-isomerase1/2 (IDI1/2), 3-ketoacyl-CoA thiolase (KCT), long chain acyl-coenzyme A dehydrogenase LCAD), malonyl-CoA decarboxylase (MCD), medium chain acyl-coenzyme A dehydrogenase (MCAD), monounsaturated fatty acids (MUFAS), mevalonate kinase (MVK), mevalonate decarboxylase (MVD), pyruvate (Pyr), phosphomevalonate kinase (PMVK), stearoyl CoA desaturase (SCD), and sterol regulatory element binding protein1,-2 (SREBP1,-2). ↑ activation, ⊥ inhibition. The figure was created with BioRender.com.

p53 has been described to play an essential role in the modulation of lipid metabolism, inhibiting FA and cholesterol synthesis and increasing FA oxidation (FAO) (Figure 5). p53 induces the transcription of lipin 1, malonyl-CoA decarboxylase (MCD), carnitine palmitoyltransferase A1,-1C (CPT1A, -1C), and carnitine acyltransferases (CATs), promoting FAO and preventing intracellular lipid accumulation [106]. Meanwhile, CPT1C translocates FA to the mitochondrial lumen [107] and lipin 1 displays phosphatidate phosphatase activity, modulating phospholipid and triacylglycerol synthesis. Lipin 1 also induces the expression of genes involved in FAO, including the PPARγ coactivator-1α (PGC-1α)/peroxisome proliferator-activated receptor α (PPARα) circuit [108] and MCD, which catalyzes the conversion of malonyl-CoA into acetyl-CoA [109].

On the other hand, p53 suppresses lipid synthesis by inhibiting the expression of SREBP-1,-2, and the SREBP1c isoform [110], contributing to the inhibition of fatty acid synthase (FASN), ACC, ACL, and the low-density lipoprotein (LDL) receptor [110,111] (Figure 5). Sung-Hwan reported that p53 transcriptionally induces the expression of ATP-binding cassette (ABCA1) transporters, decreasing SREBP2 maturation and nuclear translocation [112] and, thus, the expression of genes involved in the mevalonate pathway, such as 3-hydroxy-3-methylglutaryl-coenzyme A (HMG-CoA) synthase-1 (HMGCS1), 3-hydroxy-3-methylglutaryl-coenzyme A reductase (HMGCR), mevalonate kinase (MVK), mevalonate dehydrogenase (MVDH), isopentenyl diphosphate Δ-isomerase 1 (IDI1), farnesyl diphosphate synthase (FDPS), farnesyl diphosphate farnesyl transferase (FDPFT), and squalene epoxidase (SQLE). p53 negatively regulates mTOR and the PPP pathway, inactivating FASN [95,113].

Furthermore, p53 decreases cell proliferation by inhibiting nucleotide synthesis [114]. p53 suppresses de novo synthesis of guanosine monophosphate (GMP) and guanosine triphosphate (GTP) by inhibiting the expression of guanosine 5′-monophosphate synthase (GMPS) and amidotransferase via cyclin-dependent kinase inhibitor (p21), and inosine 5′-monophosphate dehydrogenase (IMPDH) through miR-34 induction [115,116] (Figure 5). However, in response to DNA damage, p53 induces purine and pyrimidine synthesis to facilitate DNA repair [114] via the transcriptional induction of regulatory subunits p53R2/RRM2B of ribonucleotide reductase (RNR), which reduces nucleotide diphosphates such as ADP, CDP, GDP, and UDP to produce deoxyribonucleotides (dNTPs) [117]. It has been suggested that p53 modulates PRPS and phosphoribosylformylglycinamide synthase (PFAS) activity under stress conditions.

Additionally, it has been suggested that the p19ARF/mdm2/p53 pathway has a crosstalk regulation with HIF1 to modulate cellular metabolism. p53 induces the transcription of the E3 ubiquitin ligase parkin [118], which directly binds to the transcription factor HIF1α [118]. p53 physically interacts with HIF1α, promoting the ubiquitination and degradation of HIF1α [119]. Ravi et al. reported that p53 acts as a chaperone, helping to recognize and recruit HIF1α for its ubiquitination by mdm2 [120]. In addition, p19ARF inhibits the transcriptional activity of HIF1 by sequestering to HIF1α in the nucleolus [71]. p53 decreased HIF1β levels by transcriptionally inducing miR-107, which inhibits the expression of HIF1β. Furthermore, HIF1 modulates p53. HIF1α upregulates the phosphatase1 nuclear-targeting subunit (PNUTS) mRNA, degrading mdm2 and activating p53 [121]. Interestingly, HIF1 has also been reported to inhibit p53 by increasing the levels of Mdm2,-4 [122] and blocking the transcription of p53 [123].

## 4. Role of p53 in Glioblastoma Metabolism

Mutations and deletions in tumor-suppressor genes have been frequently reported in malignant gliomas of both astrocytic and oligodendroglial lineages [124,125,126]. Alterations in p53 are the most common mutations in gliomas of astrocytic lineage, found in 50% of grade II (diffuse astrocytoma) and III (anaplastic astrocytoma) gliomas, in 25–30% of primary GBM, and in 60–70% of secondary GBM [127,128]. The most common mutations in p53 are found in the DNA-binding domain, resulting in loss-of-function, gain-of-function, and dominant-negative mutational effects for p53 [129]. Furthermore, alterations in the p53 signaling pathway have been reported in glioblastoma, including p14ARF deletions (55%), mdm2 amplification (8–11%), and mdm4 amplification (4%), in addition to mutations in p53 itself [130,131]. Overall, the ARF/mdm2/mdm4/p53 pathway is disrupted in 87% of GBM, suggesting it plays a key role in this tumor. On the other hand, mutations that result in p53 inactivation are strongly associated with a more aggressive glioblastoma phenotype, with enhanced proliferative, migratory, and invasive capacity [130,132,133], as well as increased resistance to apoptosis and chemotherapy [134]. Despite the high mutation frequency of TP53 and its regulators (ARF-mdm2/4), it has not been associated with survival and/or prognosis in glioblastoma [133,135,136]. 

Other p53 regulators have been suggested to play a role in glioma cells. For instance, p53 is regulated at a post-translational level by NAD+ synthase nicotinamide mononucleotide adenylyltransferase (NMNAT), which enhances p53 deacetylation and PARylation via the formation of a NMNAT/p53/poly (ADP-ribose) polymerase 1 (PARP-1) complex and decreased levels of (NAD+ dependent) SIRT1 deacetylase [137], which inhibits the pro-apoptotic function of caspase-3 upon cisplatin treatment in a glial neoplastic model [138]. NMNAT catalyzes the last step in NAD+ production [138]. The overexpression of Ring finger protein 216 (RNF216) (an E3 ubiquitin ligase) in human GBM tissues promoted p53 ubiquitination and degradation, preventing apoptotic cell death induced by radiation and DNA damage in glioblastoma cells [139].

It has been established that p53 expression is regulated at a transcriptional level. IDH mutants (R132Q/R132H) have shown a higher production of 2-hydroxyglutarate, promoting the stability of HIF2α, which activates miR-380-5p expression, inhibiting p53. Elevated mutated IDH1 (R132H) levels are associated with the downregulation of p53 in human glioma biopsy samples. miR-141-3p has been suggested to negatively regulate p53 expression in glioma, correlating with progression, malignity, and temozolomide resistance [140].

In addition, disruptions in the transactivation capacity of p53 have been reported [141,142]; for instance, the nuclear protein Bcl2-like 12 (Bcl2L12), overexpressed in primary glioblastoma cells, binds to p53, inhibiting its transactivation potential, suppressing apoptosis via downregulation in the genic transcription of Bax, p21, phorbol-12-myristate-13-acetate-induced protein 1 (Noxa), and Puma [142], and contributing to therapeutic resistance in glioma cells. Thus, treating GL15 glioma cells with Lonidamine inactivates the PI3K/AKT pathway, leading to the mitochondrial translocation of p53, the subsequent permeabilization of the mitochondrial membrane, and the release of pro-apoptotic factors like AIF, Bax, Noxa, and Puma, triggering apoptotic cell death [143]. Mitochondrial p53 binds to and inhibits anti-apoptotic proteins like Bcl-2 and Bcl-xL, inducing apoptotic cell death via mitochondrial permeabilization [144]. This evidence points to the various mechanisms underlying the capacity of glioma cells to inactivate the tumor suppressor p53, favoring their survival, proliferation, migration, invasion, resistance to cell death, and metabolic reprograming.

The capacity of p53 to control key metabolic features in glioma cells is well established. p53 binds to the LIM homeodomain (LIM-hd) transcription factor 9 (LHX9), inhibiting the expression of phosphoglycerate kinase 1 and lowering lactate levels, thereby decreasing glioma cells migration and invasion [145,146]. LHX9 expression levels are significantly reduced in human glioma tissues [146] and are associated with poor survival in glioma patients [145,146] (Figure 3). 

p53 has been suggested to inactivate HIF1 and, as a result, inhibit the expression of PDHK, promoting metabolic reprogramming and the antineoplastic effects of Dichloroacetat (DCA) on glioma cells [147]. DCA inhibits proliferation in glioblastoma stem cells (GSCs) and glioma cells in vitro, as well as tumor growth and angiogenesis in vivo, via p53 [147]. DCA, in combination with irradiation or etoposide, promotes apoptosis via Bax in GSCs in vitro and in vivo via p53 and FOXO3, resulting in the overexpression of Noxa, Bad, and Puma, which facilitates Bax-induced apoptosis [148].

Similarly, the activation of the complex I subunit NADH dehydrogenase (ubiquinone) 1α subcomplex and subunit 10 of complex III (INDUFA10) of the ETC decreases reactive oxygen species (ROS) generation and p53 activation, inhibiting the glycolytic switch and GLUT1,-3, PDHK1, and LDHA expression, thus reducing cell proliferation in neural stem cells and tumor formation upon transplantation into mouse brain [149] (Figure 3). Wang et al. reported that silibinin inhibits glycolysis in glioma cells by depleting HX2, PFK, and PKM2, thereby decreasing the levels of G6P and pyruvate, promoting autophagy. Meanwhile, autophagy induces p53 phosphorylation and ROS accumulation by depleting cysteine and GSH levels and downregulating glutamate/cystein xCT antiporter, with the ensuing mitochondrial translocation of Bcl-2/adenovirus E1B 19-KDa interacting protein 3 (BNIP3) [150]. In turn, BNIP3 induces mitochondrial damage, triggering ROS production and the translocation of AIF from mitochondria into the nucleus. Finally, AIF leads to caspase-independent apoptosis [150].

p53 also participates in tumor suppression by inducing glutaminase (GLS2) expression in glioma cells (Figure 3). GLS increases glutamate, α-ketoglutarate, and ATP levels, replenishing GSH levels via glutamate production, lowering ROS levels, and protecting cells from oxidative stress [103]. Furthermore, p53 positively regulates the expression of antioxidant proteins such as parkin, glutathione peroxidase-1 (GPX1), sestrins1,-2, TIGAR, and aldehyde dehydrogenases (ALDH4) [151,152,153,154,155], reducing the generation of ROS, which cause DNA damage and mutation, inducing tumor initiation and progression [114]. Restoring GLS2 expression in T96G glioma cells inhibited cell proliferation, survival, and migration; furthermore, it increased cell sensitivity to alkylating agents like temozolamide (TMZ) and carmustine by downregulating O6-methylguanine-DNA methyltransferase (MGMT) [156,157].

In addition, Culturing glioma cells under nutrient deprivation induces the expression of malate dehydrogenase 1 (MDH1), which promotes stabilization, nuclear localization, and transcriptional activity of p53 to maintain energy homeostasis, inducing cell cycle arrest and apoptosis [158]. A dysregulation of MDH1 and overexpression of Mdm2 in glioblastoma may result in tumor formation and tumor growth by inactivating p53 [158] (Figure 3). The lack of MDH1 in primary glioblastoma cells can impair mitochondrial NAD reduction through the aspartate-malate shuttle [159]. This shuttle is indispensable for the net transfer of cytosolic NADH into mitochondria to maintain a high glycolysis rate and sustain a rapid tumor cell growth [160]. Mutual regulation between p53 and the malic enzyme-2 (ME2) has been reported [161]. Malic enzymes catalyze the oxidative decarboxylation of L-malate into pyruvate and either NADH or NADPH. Its expression is positively associated with WHO tumor grade in human primary gliomas, suggesting that ME2 could be a predictive biomarker in human gliomas [162]. Chiao-Pei et al. reported that ME2 negatively regulates p53 function in glioblastoma cells and might be involved in GBM cell growth, proliferation, metabolism, and invasion [162]. ME2 depletion strongly induces p53 stability and senescence in tumor cell lines, while overexpression of this enzyme delays the process [161]. It has been suggested that the activation of p53 upon ME1 and ME2 inhibition is required to decrease Mdm2 levels and ROS-induced AMPK activation, respectively [161]. Elevated p53 concentrations reduce the expression of ME1, -2, and -3 by directly binding to response elements in these genes [161]. 

Paradoxically, p53 has been reported to promote cell survival. In this sense, TIGAR, a p53 target gene often overexpressed in glioblastoma, protects glioma cells under hypoxic conditions against oxidative stress death, inhibiting the glycolytic pathway by reducing F2,6BP levels and diverting Glu uptake to PPP in the presence of TKTL1, increasing NADPH and GSH synthesis [163] (Figure 4). Conversely, under physiologic oxygen conditions (5% O_2_), TIGAR reduces Glu uptake and lactate levels, enhancing mitochondrial respiration and ATP production, as well as PPP activity, improving glioma cell survival through a more efficient energy production and ROS detoxification [163]. TIGAR knockdown results in radiosensitization of U87 MG and T98G glioma cells, showing a senescent phenotype and a failure in DNA repair by ROS generation [164]. TIGAR induces resistance to death under tumor microenvironment conditions in glioma cells with wild-type (wt) p53, limiting glycolysis and promoting OXPHOS by inducing SCO2 synthesis [165]. The inactivation of p53 and/or SCO2 increases glycolysis and impairs mitochondrial metabolism, inducing rapid energy depletion, high ROS levels, and cell death [165]. 

p53 also induces PTEN expression, switching from anaerobic glycolysis to oxidative respiration by blocking the PI3K/AKT/mTOR pathway [166]. The contribution of p53 to cell survival has been suggested to be an adaptive response of the cell to transient hypoxia or an inducer of cell death upon more severe or prolonged hypoxia [167]. The protective function of p53 against death has been associated with relatively low levels of its own expression [168]. p53 could owe its capacity to act as a survival promoter to the induction of genes such as p21, SLUG, and myosin VI [167]. 

In addition, p53 also plays a role in survival to Glu deprivation, which induces a p53 response via AMP-activated protein kinase [52]. Whilst glioma U87 MG (wt p53) cells are tumorigenic in nude mice, glioma T98G (mutant p53) cells are not [169]. There is evidence that p53 induces resistance to methylating (TMZ) and chloroethylating (nimustine, carmustine, or lomustine) anticancer drugs in glioma cells, modulating the transcription of DNA repair genes such as O-6 methylguanine-DNA methyltransferase (MGMT), an enzyme that removes methyl and chloroethyl groups from the O6-position of guanine [170,171]. Loss of MGMT by methylation of its promoter could result in a favorable response to alkylating drugs [172].

p53 also contributes to glioma cell survival by upregulating the transcription of enzymes involved in mevalonate metabolism, such as 3′-hydroxy-3′-methylglutaryl-CoA reductase, MVK, FDPS, FDFT, geranylgeranyl transferase 1α, and LDL receptor, independently from SREBP1 and SREBP2, promoting cholesterol de novo synthesis [173]. Kambach et al. reported a link between upregulation of the mevalonate and cholesterol pathways with poor prognosis in glioblastoma patients [174]. Culturing glioma cells at a high cell density stimulates oxygen consumption, aerobic glycolytic processes, and PPP to generate substrates such as pyruvate, acetyl-CoA, ribulose-5P, ribose-5P, and NADPH to synthetize cholesterol, decreasing the levels of ROS, Krebs cycle intermediates (oxaloacetate, citrate, alpha-ketoglutarate, succinate, malate, and fumarate), and ATP [174]. Pharmacological inhibitors of cholesterol biosynthesis, such as ketoconazole, induce cell death in glioblastoma tumor-initiating cells [174]. Avasimibe, an acyl-coenzyme A:cholesterol acyltransferase-1 (ACAT1) inhibitor, promotes cell cycle arrest in the G0/G1 and G2/M phases and mitochondrial apoptosis in glioma cells [175].

Thus, wt p53 can either protect cells from death or promote apoptosis. Therefore, a better understanding of the role of p53 in the human biology, as well as in the regulation of cell life, death, and metabolic adaptation, may facilitate the identification of novel cellular targets.

## 5. Role of HIF1 in Cell Metabolism

Among the various factors known to promote tumor growth and metastasis in solid tumors comes hypoxia. It is associated with poor prognosis in patients due to incomplete or dysfunctional vascularization [176]. Hypoxia causes a drastic decrease in energy production by cells. The lack of O_2_, required for OXPHOS, stimulates metabolic reprogramming driven by HIF1. This protein induces the transcription of genes regulating survival, migration, invasiveness, angiogenesis, and metabolism in tumor cells [176].

The transcriptional factor HIF1 Is a heterodimeric protein consisting of subunits HIF1α and HIF1β. HIF1β is constitutively expressed and translocates to the nucleus regardless of the O_2_ concentration [177]. Meanwhile, HIF1α is found in the cytoplasm; its stability is regulated by intracellular O_2_ levels due to the presence of an O_2_ concentration-dependent degradation domain (ODD). Under normal oxygenation conditions (normoxia), HIF1α degradation is mediated by prolyl hydroxylase domain (PHD) enzymes. This promotes its binding to the Von Hippel–Lindau protein (pVHL), an E3 ubiquitin ligase that leads to its degradation via the ubiquitin-proteosome system [178]. The transcriptional activity of HIF1α can be inhibited by a factor-inhibiting HIF1 (FIH), which inhibits the recruitment of the coactivator CREB-binding protein (CBP)/p300 [179]. In addition to oxygen, PHDs and FIHs require Fe^2+^, 2-oxoglutarate (2-OG), and ascorbate as substrates for hydroxylation [180]. Succinate and fumarate accumulation due to the inhibition of succinate dehydrogenase (SDH) and fumarate hydratase (FH), respectively, inhibits PHDs, leading to the stabilization and activation of HIF1α [181]. Under hypoxia, PHD and FIH activity decreases, as both enzymes are oxygen-dependent; this reduction in HIF1α hydroxylation favors its nuclear translocation and stabilization. Furthermore, ROS can inhibit PHD and FIH activity, oxidizing Fe^2+^ to Fe^3+^ [182]. Nuclear HIFα forms a complex with its homodimer HIF1β. This complex binds to hypoxia response elements (HREs) located in the 5′ promoter region of its target genes, promoting their expression [183,184].

HIF1 induces the expression of most genes involved in glycolysis, including GLUT1 and GLUT3, and glycolytic enzymes hexokinases 1,-2 (HX 1,-2), glucose phosphate isomerase (GPI), PFK1, aldolase-A, -B (ALDO)-A, -B, triosephosphate isomerase (TPI), glyceraldehyde 3-phosphate dehydrogenase (GA3PDH), PGK1, phosphoglycerate mutase 1 (PGAM 1), enolase 1 (ENO1), PKM2, and LDHA [182,185,186]. Thus, HIF1α induces Glu uptake and increases lactate production from pyruvate [182,185,186]. The excess lactate and H^+^ in hypoxic cells are exported by cytosolic pH regulators such as MCT1, -4, Na^+^/H^+^ exchanger (NHE1), and carbonic anhydrase IX (CA9), all of which are activated by HIF1α under hypoxia [187,188] (Figure 2). The result is intracellular alkalinization and extracellular acidification, which promote proliferation, angiogenesis, invasion, and metastasis via degradation of the extracellular matrix [189].

Metabolomic analysis showed that HIF1 also induces PPP by positively regulating the expression of G6PDH and the transketolases TKT and transketolase-like protein 2 (TKTL2) [190,191], thereby promoting ribose and NADPH synthesis, as well as intermediates of the glycolytic pathway such as F6P and glyceraldehyde 3-phosphate (GA3P) from xylulose 5-phosphate (X5P) [192] (Figure 3).

Under hypoxia, HIF1 induces a shift from oxidative to glycolytic metabolism, decreasing TCA cycle activity and, consequently, flux to the CTE (Figure 2) [193,194]. HIF1 induces the expression of PDHK1, which inhibits PDH, reducing the levels of metabolites such as citrate, α-KG, succinate, fumarate, and malate [195]. PDH converts pyruvate into acetyl-CoA. Typically, acetyl-CoA feeds the TCA cycle to generate intermediates along with NADH and FADH2, which are essential for mitochondrial respiration [193]. Furthermore, HIF1 activation reduces the activity of the α-ketoglutarate dehydrogenase (α-KGDH) complex by inducing transcription of the E3 ubiquitin ligase seven in absentia homolog 2 (SIAH2), which promotes the degradation of the oxoglutarate dehydrogenase subunit in the α-KGDH complex [196]. α-KGDH catalyzes the production of succinyl-CoA and NADH from α-KG and NAD+, respectively [197]. In response, HIF1 activates to decrease mitochondrial respiration and electron leakage from the ETC [193,194]. 

When O_2_ tension is low, HIF1 also prevents ROS formation, modulating the function of the ETC by transcriptionally activating the dehydrogenase [ubiquinone] 1 alpha subcomplex and 4-like 2 (NDUFA4L2), a component of complex I, reducing the activity of complex I and curbing ROS production [198]. HIF1 activates the components of complex IV through the expression of the cytochrome-c-oxidase (COX) subunit 4-2 (COX4-2) isoform and the mitochondrial protease LON, which degrades COX4-1. Then, COX4-2 substitutes for COX4-1 in cytochrome c oxidase (complex IV), resulting in a more efficient electron transfer to oxygen without increasing ROS levels [194,198], as COX4-1 expression correlates with elevated complex IV activity and increased ROS production [199]. COX4-1 is involved in the formation of mitochondrial supercomplexes containing CTE complex IV, thus promoting an increased rate of respiration and an OXPHOS phenotype in radioresistant cells [199]. Oliva et al. reported a strong association of COX4-1 levels with poor prognosis in cancer patients, whereas higher COX4-2 concentrations were not correlated in such a way [200].

Furthermore, HIF1 regulates the activity of the ETC by transcriptionally regulating miR-210 [201], which inhibits mRNA synthesis for the subunit D of the SDH complex II, decreasing complex II activity and the accumulation of succinate, thereby destabilizing HIF1α [202]. The iron-sulfur cluster assembly proteins (ISCU1/2) (complex I subunits), NDUFA4, and COX10 (complex IV subunits) are also targets of miR-210 [202,203,204].

HIF1α can suppress β-oxidation by positively regulating the expression of fatty acid binding proteins 3 and 7 (FABP 3/7) and by suppressing the expression of medium/long-chain acetyl-CoA dehydrogenase (MCDA/LCAD) and activating PGC-1α, a transcriptional coactivator of CPT1A [205,206,207,208]. HIF2α also inhibits CPT1A transcription [209]. By activating FABP 3/7 and repressing CPT1A, HIF1/2 promotes lipid droplet (LD) formation [205]. Furthermore, HIF1 induces the expression of the adipose differentiation-related protein (ADRP), a factor required for LD formation and the uptake of long-chain FAs [210,211] (Figure 4).

HIF1 induces FA synthesis by promoting stearoyl-CoA desaturase (SCD) expression [212]. It also induces FASN transcription via the Akt/HIF1/SCREB1 pathway and possibly the expression of ACL [213,214]. HIF1 also induces FA synthesis from glutamine, which is converted to α-KG, which then undergoes reductive carboxylation by isocitrate dehydrogenase 1 and -2 to produce citrate. Citrate is then converted to acetyl-CoA, a precursor of FA [215].

Additionally, it has been suggested that HIF1 and HIF2alfa have crosstalk regulation with c-Myc to modulate cellular metabolism. HIF2α increases c-Myc transcriptional activity, promoting the formation of the c-Myc/Max complex [216], which directly induces HIF2α expression. c-Myc increases the expression of the glutamine transporters solute carrier family 1 member 5 (SLC1A5) and SLC7A2, as well as GLS and LDHA [217] (Figure 2). c-Myc and HIF1 cooperatively activate PDHK1 to inhibit mitochondrial respiration [218,219]. c-Myc induces pVHL SUMOylation, increasing its accumulation and inhibiting HIF1α degradation [220]. In contrast, HIF1α represses c-Myc activity by binding to Max and preventing the c-Myc/Max transcriptional complex from forming [221]. HIF1α also induces proteasomal degradation of c-Myc under hypoxia [222].

Other factors are known to regulate HIF1 under hypoxia. Kaidi et al. reported that, under hypoxia, the binding of β-catenin with HIF1 increases the transcriptional activity of HIF1 by inhibiting the formation of the β-Catenin/TCF4 complex, which promotes the adaptation of cancer cells to hypoxia. PKM2 is a transcriptional coactivator for HIF1, amplifying HIF1 activity via a positive feedback regulation and thereby promoting cancer progression. In addition, dimeric PKM2 activates signal transducer and activator of transcription 3 (STAT3) by phosphorylation at Tyr705, and STAT3 induces the transcription of HIF1α. On the other hand, ERK stimulates the transcriptional activity of HIF1 by phosphorylating CBP/p300, allowing the formation of a p300/HIF1α complex.

## 6. Role of HIF1 in Glioblastoma Metabolism

Several studies have shown that hypoxia and HIF1 promote the initiation and progression of carcinogenic processes; they also induce resistance to chemotherapy and radiation, and recurrence in GBM [223]. Tumor hypoxia has been associated with poorer clinical outcomes in GBM patients [223]. Hypoxia seems to interact with HIF1 to create an ideal microenvironment for the growth of brain tumor-initiating cells (BTICs), which contribute to high intratumor heterogeneity and tumor recurrence [224]. Increased levels of HIF1 expression and transcription have been found in peripheral, avascular, and hypoxic areas of biopsies from GBM patients with respect to lower-grade biopsies, confirming that hypoxia plays a key role in the development and aggressiveness of glioma [225]. MCT1 and NHE1 expression is higher at the leading edge of tumors in GBM patients and in a rat glioma model; in contrast, MCT4 is upregulated in the perinecrotic tumor center, along with HIF1α, LDH, and CA9 [226,227,228]. Furthermore, survival is lower in patients overexpressing HIF1α that underwent surgery for GBM [229].

Hypoxia induces metabolic reprogramming in GBM cells [224], which favors biomass production to sustain tumor proliferation, synthesizing metabolic intermediates via glycolysis activation [35]. Sanzey et al. demonstrated that patient-derived stem cells and glioblastoma cells subjected to severe hypoxia (0.1% O_2_) overexpress glycolytic enzymes such as HX2, PFK1, ALDOA, PGAM1, ENO1, -2, and PDHK1 [230]. Knockdown of each of those genes inhibited cell proliferation, inducing cell death in both stem cells and U87 glioma lines under hypoxic conditions [230]. These results suggest that glycolytic enzymes are essential for cell survival under hypoxic conditions and for tumor growth in vivo. Thus, these enzymes are potential therapeutic targets against GBM [230]. Kucharzewska et al. reported increased expression of GLUT1, -3, -4, MCT4, HX2, PFKFB3, -4, ALDO, PGK1, and PDHK3 mRNA, as well as glycolytic intermediates such as Glu, G6P, F6P, F1,6BP, and 3-phosphoglycerate [231] (Figure 2).

Hypoxia also activated the PPP to produce required molecules such as 6-phosphogluconate, ribulose 5-phosphate, X5P, R5P, and sedoheptulose 7-phosphate for the synthesis of nucleic acids and FAs, promoting the resistance and progression of glioma cells by activating non-glucose metabolic pathways like the polyol pathway to increase sorbitol and fructose levels and protect cells from anoxic death [231] as fructose is an inducer of TKT activity [232]. Kathagen-Buhmann et al. reported the downregulation of G6PDH, 6-phosphogluconate dehydrogenase (PGD), and TKT, along with the overactivation of glycolytic enzymes in glioma U87 and G55 cells cultured under acute hypoxia. The result was an increased migratory capacity but decreased proliferative ability [233] (Figure 3).

Kucharzewska et al. reported decreased levels of metabolites produced by the TCA cycle in U87 glioma cells cultured under hypoxic conditions, along with significantly increased concentrations of 2-hydroxyglutarate (2HG), an oncometabolite [231] synthesized from α-KG by the IDH1 and IDH2 mutant forms expressed in some gliomas and secondary GBMs [234]; decreased levels of α-KG negatively regulate the stability of HIF1α [235]. It has been suggested that hypoxic U87 glioma cells produce α-KG from glutamine, a reaction catalyzed by glutamic pyruvate transaminase 2 (GPT2), given the increased intracellular concentrations of glutamine and glutamate [234]. GTP2 is induced by HIF2α in human glioma cells under hypoxic stress, and GPT2 inhibition decreases cell proliferation and migration in vitro and in vivo [16]. GTP2 produces α-KG and alanine from pyruvate and glutamate [236] (Figure 2).

Kucharzewska et al. also reported decreased cholesterol synthesis in hypoxic U87 glioma cells exposed to elevated levels of squalene, lathosterol, and lanosterol, as well as increased rates of degradation of 3-hydroxy-3-methylglutaryl-CoA reductase (HMGCR), the enzyme that catalyzes the rate-limiting step in the cholesterol synthesis pathway, via the ubiquitinase insulin-induced gene 2 (Insig2) [231] (Figure 4). They also suggested that hypoxia does not deplete metabolites (carnitine, acetylcarnitine, propionylcarnitine, and butyrylcarnitine) involved in FA β-oxidation, but it increases the levels of FAs and their metabolites (palmitic acid arachidic acid and prostaglandin E2), triacylglycerols (glycerol 3-phosphate), and phospholipids (choline, choline phosphate, glycerophosphorylcholine, and glycerophosphoethanolamine) in glioma U87 cells [231] (Figure 4). These authors also showed that prolonged hypoxia results in a reserve of post-translationally modified amino acids and dipeptides, suggesting that glioma cells accumulate free amino acids to meet energy demands that cannot be sufficiently supplied by glycolytic flux [231]. 

HIF1 is regulated by several signaling pathways during metabolic reprogramming. Blum et al. reported that hypoxia promotes HIF1α stabilization, along with the overexpression of CA9, MCT4, NHE1, GLUT1, and GLUT3 in U87MG and U251 glioma cells. In contrast, HIF1α inhibition reduced CA9 expression in hypoxia, as well as cell migration and monocyte adhesion in both U87 and U251 cells [237]. These authors suggested that HIF1α stabilization and CA9 expression are mediated by EGFR/STAT3 signaling [237]. A positive association between CA9 and HIF1α expression was found in GBM patients. Higher CA9 expression is associated with a poorer prognosis [237], malignancy [238], and a lower response to chemo- and radiotherapy [239]. Jing-Huei et al. reported that Nodal, a TGFβ family member, promotes a shift in Glu metabolism by inducing HIF1 transcription, which increases GLUT1, HX2, and PDHK expression, as well as the phosphorylation (inactivation) of PDH, favoring Glu uptake, increasing lactate levels, and decreasing mitochondrial OXPHOS in human GBM8401 and U87 MG glioma cells under normoxic conditions [240]. HIF1α knockdown decreases GLUT1, HX2, and PDHK1 expression, as well as PDK1 activation, increasing mitochondrial energy metabolism [240]. In addition, the activation of HIF1 under hypoxia conditions in G06A, SDT3G, and J3TBg canine glioma cell lines induces the overexpression of HIF1α, GLUT1, and PDHK proteins, lactate levels, and higher production in the ATP levels via glycolysis that generate at mitochondrial levels, which significantly increases the cell viability from glioma cells. However, the inactivation of HIF1 with evofosfamide inhibits the GLUT1, PDHK, HIF1α, lactate, and glycolytic ATP leading to a decrease in the total ATP levels and a high rate of apoptosis of canine glioma cells in a hypoxic medium with respect control cells without treatment. Also, evofosfamide decreases the tumor volume and weight in murine glioma xenograft models, and higher levels of HIF1α, PDHK1, G6P, F6P, DHAP, 3PG, PEP, Pyr, and lactate in tumor tissue induce an increase in the overall survival in the treated animals in relation to the control group [241]. The authors suggest that HIF1 induces glycolysis by supporting the survival, growth, and progression of GBM [241]. Similar correlations among HIF1 levels and glycolysis with the survival and development of glioblastoma have been reported [242,243,244,245,246].

Inhibition of the Ras oncogene blocks survival, invasiveness, and angiogenesis in U87 MG cells via the inhibition of key kinases, including ERK, PI3K, and AKT, with the ensuing degradation of HIF1α and a downregulation of glycolytic enzymes like phosphohexose isomerase, PFKFB3, -4, Aldolase C, triose phosphate isomerase 1, glyceraldehyde 3-phosphate isomerase, phosphoglycerate kinase1, phosphoglycerate mutase1, enolase, PKM2, PDH, and LDH, as well as GLUT1, CA9, transglutaminase 2, and the vascular endothelial growth factor (VEGF) [247]. Raf/MEK/ERK signaling potentiates HIF1α transactivation activity, and the PI3K/AKT/GSK3β pathway stabilizes HIF1α [248]. Stem-like cancer cells (U87 SC) enhance their self-renewable properties under hypoxia with respect to normoxic conditions. Furthermore, they are highly resistant to doxorubicin, vincristine, and 1,3-bis(2-chloroethyl)-1-nitrosourea (BCNU), but more sensitive to glycolytic inhibitors than their U87 counterparts [249]. Meanwhile, GLUT1, HX2, and LDH expression is upregulated in U87 SC cells, but PDH is downregulated; at the same time, these cells show increased HIF1α and HIF2α stability, Glu uptake, and lactate production without affecting ATP levels [249]. U87 SC cells also show a 50% decrease in the rate of oxygen consumption due to the downregulation of succinate dehydrogenase subunit B, a subunit of complex II of the ETC, decreasing mitochondrial respiration but increasing glycolysis [249]. HIF1 stabilization could be due to either the inactivation of prolyl dehydrogenase, resulting in succinate accumulation, or indirectly by ROS generation due to a defect in mitochondrial complex II [181,250].

miR-448 has been shown to inhibit proliferation, migration, and invasion while increasing radiosensitivity in vitro and in vivo in glioma cells. It also decreased glycolysis by negatively regulating the activity of HIF1α signaling and its downstream effectors, HX1, -2, and LDHA, inhibiting lactate and ATP production [251]. HIF1α levels were decreased in glioma patients with high miR-448 levels but increased in patients with lower miR-448 expression; these differences were correlated with patient survival [251]. In contrast, miR-150 upregulation in U251 cells inhibited the expression of the tumor suppressor VHL and increased HIF1α stability. HIF1α stimulated Glu uptake, glycolysis, and lactate production by upregulating GLUT1, HXI, and LDH, promoting cell proliferation and tumor growth [252].

Heller et al. reported that hypoxia induced transketolase-like protein 1 (TKTL1) expression via HIF1 in LNT-229 glioma cells. Knockdown of TKTL1 increased Glu uptake, as well as lactate and ROS production, leading to cell death and increased sensitivity to ionizing radiation in LNT-229 cells [253]. The authors suggested that TKTL1 protects cells from death by reducing Glu requirements and increasing PPP activity, increasing NADPH+ production, GSH levels, and ROS detoxification [253]. TKTL1 has been suggested to curb ROS production by activating TIGAR [163]. Increased levels of TKTL1 mRNA expression and transcription are observed in human glioblastoma tissue and glioma cell lines [254].

Hypoxic wt-IDH glioma tissues from patients show a glycolytic phenotype due to the upregulation of PGK1, PKM2, LDHA, SLC16A3, and CA9 via HIF1. Similarly, HIF1-responsive glycolytic genes (SLC2A1, HX2, PDHK1, LDHA, SLC16A3, ENO1, and CA9) were downregulated in mutated IDH-derived brain tumor stem cells (BTSCs) with respect to IDHwt-derived BTSCs [255]. 2-HG stimulates PHD activity, leading to HIF1α degradation [256], which can prevent the glycolytic switch and decrease the elevated rate of cell proliferation typical of high-grade gliomas [255]. The overexpression of mutated IDH1 in U87 and U251 glioma cells decreased cell proliferation, migration, and invasion whilst promoting apoptosis and improving chemosensitivity to TMZ by increasing the expression of FAT atypical cadherin 1; this protein promotes ROS generation by decreasing NADPH+ levels, the activity of superoxide dismutase 2 (SOD2), and thr glutamate-cysteine ligase catalytic subunit, resulting in the upregulation of HIF1α [257]. HIF1 can induce apoptotic cell death by upregulating the expression of the pro-apoptotic protein BNIP3 and promoting p53 stability by inhibiting mdm2. Both BNIP3 and p53 inhibit pro-apoptotic proteins such as Bcl-xL and Bcl-2 [122,258,259]. BNIP3 favors the initiation of mitochondrial autophagy under hypoxia by releasing Beclin-1 from Bcl-2 and Bcl-xL, reducing oxidative metabolism [260,261]. However, Kucharzewska et al. demonstrated that prolonged hypoxia induces the expression of autophagic genes like BNIP3L, unc-51-like autophagy-activating kinase 1 (ULK1), autophagy-related gene 9 (ATG9), and vacuolar sorting protein 34 (VPS34) in U87 cells, which should protect them from hypoxic stress [234]. On the other hand, IDH mutations may favor glioma development, growth, and invasion via HIF1α induction by inhibiting PHDs [262,263].

## 7. Role of c-Myc in Cell Metabolism

c-Myc deregulation is a hallmark in human malignancies [264]. The nuclear phosphoprotein c-Myc (myelocytomatosis oncogene) is a transcription factor encoded by the c-Myc gene (a member of the MYC proto-oncogene family) [265]. The structure of c-Myc is an essential helix–loop–helix leucine zipper protein; it is activated by forming a dimer with the Max protein and is able to couple to an E-box motif (5′-CACGTG-3′) in genomic DNA [266]. However, transcription is repressed when it interacts with Msx-interacting-zinc finger 1 (Miz-1), among other core promoters [266]. c-Myc plays a crucial role in cell function as a central transcription factor that regulates approximately 10% of the genome [267]. It plays a key role in regulating the cell cycle and modulating DNA synthesis and apoptosis. In addition, it plays an essential role in regulating cell size, proliferation, metabolism, reprogramming, and differentiation [267,268,269]. Decreased c-Myc expression has been associated with quiescence [270,271]. However, constitutive expression or overexpression of c-Myc induces cell transformation [272].

The effects that c-Myc exert on glycolysis are mediated by the positive regulation of GLUT1, HX2, HX3, GPI, phosphofructokinase M 1 (PFKM1), GA3PDH, PGM, PKM2, LDHA, and ENO1 [273,274,275] (Figure 2). By upregulating these genes, c-Myc contributes directly to the Warburg effect and the ability of transformed cells to convert glucose into pyruvate; the excess intermediate metabolites and lactate are exported by MCT1, which is activated by Myc [273,276]. 

c-Myc regulates the PPP’s oxidative and non-oxidative branch phases by increasing the expression of G6PDH and TKT, respectively [268] (Figure 3).

On the other hand, c-Myc controls glutamine uptake through the transcription of transporters such as SLC3A2, SLC7A1, and SLC5A1, as well as the conversion of glutamine into glutamate via the transcriptional regulation of GLS1 and -2 [217,277] (Figure 2). c-Myc also regulates the conversion of glutamate into α-KG by positively regulating GDH, glutamate oxaloacetate transaminase (GOT), and ornithine aminotransferase (OAT) [278]. c-Myc-dependent glutamine catabolism replenishes intermediate metabolites of the TCA cycle by producing the anaplerotic substrate α-KG, but also supports the biosynthesis of amino acids, nucleotides, and lipids. Amaya et al. demonstrated that STAT3/c-Myc signaling induces the expression of the transporter SLC1A5, which increases glutamine intracellular concentrations and, as a result, produces GSH and TCA intermediates cycle like citrate, itaconate, α-KG, succinate, malate, and fumarate; this superabundance of intermediates fuels the TCA cycle, inducing OXPHOS and activating the ETC [279].

Moreover, c-Myc induced the expression of several subunits in complex I in the ETC, including the subunits A1,-A2,-B3,-B5 S1,-S6, and V1 of NADH: ubiquinone oxidoreductase (NDUF) [280] and cytochrome c [281], as well as isocitrate dehydrogenase in TCA [282] (Figure 2).

c-Myc binds to and activates SREBP1 to synergistically regulate FA synthesis, promoting tumorigenesis in vitro and in vivo (Figure 4). c-Myc induces lipogenesis by positively regulating the transcription of SREBP1, ATP citrate lyase (ACL), acetyl-CoA carboxylase A (ACCA), FASN, fatty acid elongases (ELOVL2 and ELOVL6), and stearoyl CoA desaturase (SCD1) [283]. In contrast, c-Myc downregulates the expression of genes involved in β-oxidation, including CPT1A, CPTIB [283]. c-Myc induces the synthesis of mRNA coding for genes involved in the synthesis of cholesterol and steroid hormones, such as FDFT1, squalene monooxygenase (SQLE), lanosterol synthase (LSS), CYP5IAI, NAD(P)H steroid dehydrogenase-like (NSDHL), 17-beta-hydroxysteroid dehydrogenase type 7 (HSD17B7), emopamil-binding protein (EBP), sterol-C5-desaturase/lathosterol oxidase (SC5DL), and sterol-∆7-reductase (DHCR7) [283].

c-Myc also regulates the expression of genes involved in nucleotide biosynthesis, including inosine monophosphate dehydrogenase-1, -2 (IMPDH-1, -2), and the phosphoribosyl pyrophosphate synthetase 2 (PRPS2) gene [268,284,285] (Figure 3). Wang et al. showed that c-Myc also regulates the expression of phosphoribosyl pyrophosphate amidotransferase (PPAT), formylglycinamide synthase (PFAS), phosphoribosyl aminoimidazole carboxylase (PAICS, bifunctional), 5-aminoimidazole-4-carboxamide ribonucleotide formyltransferase/IMP cyclohydrolase (ATIC, bifunctional), adenylosuccinate synthase (ADSS), adenylosuccinate lyase (ADSL), and guanine monophosphate synthase (GMPS), enzymes involved in purine synthesis [286,287].

c-Myc regulates pyrimidine synthesis de novo by positively regulating carbamoyl phosphate synthetase, aspartate transcarbamylase, dihydroorotase (CAD, trifunctional), dihydroorotate dehydrogenase (DHOD), nucleoside diphosphate kinase (NDPK), CTP synthase 1,2 (CTPS), and thymidylate synthase (TS), which participate in uridine, cytosine, and thymine synthesis [288,289].

Additionally, it has been suggested that c-Myc has crosstalk regulation with HIF1 for the modulation of cellular metabolism. A synergistic effect has been reported between deregulated c-Myc and HIF1 to promote hypoxic adaptation in cancer cells via the transcription of VEGF, HX2, and PDHK1, all of which are involved in metabolic reprogramming and angiogenesis [290]. On the other hand, HIF1 induces the inactivation of c-Myc under hypoxia, as elevated c-Myc levels induce apoptosis [291]. HIF1 inhibits the transcriptional activity of c-Myc, either by direct interaction and transcriptional regulation of Mxi-1, a c-Myc antagonist [216,291,292], or by displacing c-Myc in regulatory regions of target genes [293]. Li et al. reported that c-Myc promoted HIF1α stability, preventing its proteasomal degradation [294].

## 8. Role of c-Myc in Glioblastoma Metabolism

In most cases, c-Myc is activated after gene translocation or amplification, enhanced protein translation, or stability leads to the overexpression of a protein structurally similar to the wild type [264]. Previous reports indicate that c-Myc is frequently upregulated or amplified in glioma cells [295]. Faria et al. found that the nuclear expression of the c-Myc phosphoprotein is correlated with malignancy grade in human astrocytic gliomas, so c-Myc overexpression is higher in grade-III and -IV tumors [296]. Orian et al. reported that c-Myc expression in human glioma biopsy samples significantly increases according to the histopathological grade of astrocytoma cases, with 33% expression in grade III and 76% in grade IV tumors [297]. In addition, the increase in c-Myc expression levels were higher in recurrent glioblastoma than in newly diagnosed GBM [298]. There is evidence that c-Myc activation is involved in the progression of IDH1-mutant glioma [299]. c-Myc mRNA expression is 80.6% and 78% higher in primary and secondary glioblastoma biopsy samples with respect to normal brain samples [300,301].

c-Myc expression in glioma cells is regulated at an epigenetic level. A correlation was reported between the overexpression of histone demethylases (KDM4C) and c-Myc transcripts in human glioblastoma tissue [302]. In glioblastoma cells, KDM4C binds to the promoter region of c-Myc to increase the demethylation of histone 3 (H3) at Lys9 (H3K9me3), promoting c-Myc expression and tumorigenesis [302]. KDM4C also inhibits the pro-apoptotic activity of p53 by inducing its monodemethylation at Lys372 (p53K372me1), increasing its instability [302,303]. PKM2 joins to and phosphorylates at Thr11 in H3, inducing the release of histone deacetylases (HDAC) from the H3 promoter, the subsequent acetylation of H3 at Lys9, and Myc transcription [304]. Phosphorylation levels of H3 at Thr11 correlate with the expression of nuclear PKM2, tumor grade, and survival in glioma patients [304]. Upon phosphorylation at Ser 37 by ERK in response to EGFR activation, PKM2 is translocated to the nucleus, where it acts as a coactivator of β-catenin, promoting the expression of c-Myc [305]. Phosphorylation of PKM2 at Ser37 correlates with EGFR and ERK1/2 activity in human GBM [305].

As in other malignancies, c-Myc activity induces a metabolic shift in glioblastoma. Masuai et al. found that the overexpression of EGFRvIII and mTORC2 controls glycolytic metabolism and proliferation in glioma U87 cells via c-Myc [306] (Figure 3). The authors suggested that mTORC2 promotes the phosphorylation of class IIa histone deacetylases, leading to the acetylation of the transcriptional factor FOXO and the release of c-Myc from miR-34-c-dependent suppression, resulting in higher GLUT1, HX2, LDHA, and PDHK1 mRNA levels [306]. FOXO negatively regulates c-Myc RNA stability and translation by increasing miR-34-c and miR-145 levels [55,307]. On the other hand, higher acetylated FOXO and c-Myc levels are correlated with poorer prognosis in GBM patients [306]. In addition, histone deacetylase inhibitors (vorinostat, panobinostat, and romidepsin) inhibit glycolysis in human GBM models by transcriptionally repressing c-Myc, with a shift to OXPHOS, activation of β-oxidation, and inhibition of FA synthesis [308].

EGFRvIII favors glycolytic metabolism, increasing the levels of GLUT1, -3, HX2, and PDHK1, led by c-Myc activation due to delta Max, an alternative splicing of Max promoted by the heterogeneous nuclear ribonucleoprotein 1 (hnRNPA1) [309]. A strong correlation has been reported between the expression of hnRNPA1 and that of GLUT 1,-3, HX2, and PDHK1 in GBM tissues samples, leading to significantly lower patient survival [309]. Li et al. showed that silencing karyopherin α2 (KPNA2) in U87 and U251 GBM human cells decreases cell proliferation and invasiveness by downregulating the expression and translation of GLUT1, MCT1, PFK2, PKM1, programmed death-ligand 1 (PDL1), GLS, and SLCA5 mRNA and decreasing 2-deoxyglucose uptake and lactate production; meanwhile, the OXPHOS rate was moderately inhibited [310]. The authors proposed that karyopherin (KPNA2) induced the nuclear translocation of E2F1, where E2F1 regulates the levels of c-Myc mRNA [310]. Interestingly, Linc01060 (long noncoding RNA) isolated from exosomes derived from glioma stem cells under hypoxia were found to promote progression in glioma cells by facilitating aerobic glycolysis, inducing HX, PGK1, and LDHA expression, as well as Glu uptake, and increasing lactate and ATP levels [294]. Linc01060 levels were higher in exosomes isolated from GBM patients with respect to healthy patients [294]. Linc01060 interacted with and stabilized the transcription factor myeloid zinc finger 1 (MZF1), facilitating the nuclear translocation of MZF1 and promoting c-Myc transcriptional activities; c-Myc enhanced HIF1 stability, upregulating Linc01060 transcription [294]. The activity of the Linc01060/MZF1/c-Myc/HIF1α axis was higher in human GBM biopsy samples than biopsies from low-grade glioma patients, correlating with tumor grade and a poorer clinical prognosis [294]. Meanwhile, inhibiting c-Myc with omomyc (a dominant-negative c-Myc miniprotein) decreased HIF1α binding to target promoters, strongly suppressing the expression of a subset of genes, inhibiting hypoxia-dependent glycolytic reprogramming, and increasing oxidative phosphorylation [26]. In addition, treating tumorspheres derived from GBM patients with inhibitors of nicotinamide phosphoribosyl transferase (NAMPT), a NAD+ salvage enzyme, resulted in c-Myc overexpression, which mediated cytotoxicity by apoptosis in tumorspheres, inhibiting glycolysis in the NAD+-dependent GA3PDH step, decreasing the enzymatic activity of HX2, PKM2, and LDHA, as well as the levels of NAD+, pyruvate, and lactate [311]. NAMPT inhibition increased survival in mice intracerebrally xenografted with biopsy samples from GBM patients overexpressing c-Myc [311]. c-Myc has been reported to induce NAMPT mRNA [312], and c-Myc knockdown inhibits the malignant capacity of glioma stem cells [313] (Figure 4).

c-Myc controls glutamine metabolism, and in turn, the TCA cycle (Figure 3). The increased amplification of c-Myc in Glu-deprived SF188 glioma cells induces the overexpression of glutamate dehydrogenase, which enables glioma cells to use glutamine to survive, increasing α-KG levels and ultimately OA, citrate, pyruvate, and Ac-CoA through the TCA [314]. c-Myc also regulates glutaminolysis since glutamine is a nitrogen source for purine synthesis, as well as a source of metabolic intermediates to feed the TCA in BTICs [286] (Figure 3). Glutamine levels are elevated in GBM [141] due to the overexpression of glutamine synthetase (GS), which produces glutamine from glutamate and ammonia [315].

c-Myc also regulates the mevalonate pathway in glioma. Human BTICs require significant c-Myc overexpression for self-renewal and tumor growth [313] (Figure 5). c-Myc regulates mevalonate metabolism in patient-derived BTICs by binding to the promoter regions of HMGCR, PMVK, MVK, MVD, IDI1, and FDPS [316]. Inhibiting the mevalonate pathway with shHMGCR and/or statin (an HMGCR inhibitor) reduced cell proliferation, self-renewal, and tumorigenicity in RAS/ERK-dependent BTICs [316]. The mevalonate pathway sets a positive feed-forward loop to activate c-Myc signaling by inducing miR-33b [316]. HMGCR levels were associated with poor prognosis in GBM patients [316].

c-Myc modulates purine synthesis in GSCs. c-Myc silencing by shRNAs in BTICs significantly reduced the transcription and translation of PRPS1, PPAT, ADSS, ADSL, GMPS, and IMPDH1 involved in the purine biosynthetic pathway; as a result, the production of IMP, AMP, and GMP also decreased (Figure 4). The overexpression of PPAT, ADSS, ADSL, and IMPDH1 correlated with lower survival in GBM patients [286]. 

## 9. Therapeutic Strategies against the Transcriptional Factors p53, HIF1, and c-Myc in Glioma

One of the hallmarks of GBM is metabolic reprogramming, the development of drugs that modulate the different metabolic pathways represents a therapeutic strategy for the benefit of patients. Drugs targeting the transcriptional factors p53, HIF1, and c-Myc that modulate cellular metabolism in glioma cells are described below.

### 9.1. p53 Inhibitor Drugs

#### 9.1.1. Drugs Activating p53 by Blocking the mdm2/p53 Binding

Nutlins (cis-imidazoline analogous) are small molecules that inhibit mdm2 and the ensuing formation of the p53-mdm2 complex, favoring p53 stability and activation [317] (Table 1). Nutlin3a promotes cell cycle arrest, mitochondrial apoptosis, and cell senescence in U87MG (p53WT) glioma cells but not in p53-defective T98G cells [318]. Nutlin3a synergizes the pro-apoptotic and anti-invasive effects of TMZ by activating the p53 pathway, both in vitro and in mice intracranially transplanted with human glioma cells [317]. A combination of archazolid (a V-ATPase inhibitor) and nutlin3 synergistically reduced metabolic activity in vitro and tumor growth in U87MG xenograft mice [319] via p53. This treatment induced synergistic effects in breast (MCF7) cancer cells, inhibiting glycolytic activity by decreasing Glu uptake, GLUT1 and TIGAR expression, and ATP concentrations; it also synergistically increased the levels of RB, insulin-like growth factor-binding protein 3 (IGFBP3), and pAKT, leading to apoptosis by increasing Bax levels, caspase-9 activity, and PARP hydrolysis [319]. Nutlin3 also reduced the transcription of pyruvate carboxylase, disrupting the TCA cycle and reducing OA, NADPH, and ATP levels [320]. 

RG7112 and RG7388, other nutlin analogous with higher potency than nutlin3, exhibit antitumor effects on cancer cells via mdm2 inhibition. RG7112 induces apoptosis in human glioma (TP53wt, mdm2amp) cells by increasing p53 and p21 levels and downregulating Ki67 [321]. RG7112 also reduces tumor growth and increases survival in glioma (mdm2amp) subcutaneous and orthotopic xenograft mice [321]. RG7388 has shown higher potency and selectivity than RG7112 to inhibit mdm2 [322]. RG7388 decreased cell proliferation and clonogenicity in wt-p53 A172, U87MG, and glioma-initiating cells [323]. Furthermore, RG7388 plus radiotherapy has shown synergistic effects in reducing clonogenicity and arresting cell cycle in the G1 phase on wt-p53 glioma cells [323]. A phase I/II study (NCT03158389), which included co-treatment with RG7388 and radiotherapy on patients with newly diagnosed GBM with MGMT promoter demethylation, is ongoing to determine tolerability, PFS-6, and OS [324].

Another small molecule that inhibits the mdm2 binding p53 is AMG232. Upon administering AMG232 before surgery in patients with wt-p53, recurrent GBM showed adequate tumor penetration and the induction of p21 expression [325]. AMG232 is more potent than RG7388 in inhibiting mdm2 [326,327]. Moreover, this compound has been evaluated in phase I study in patients with advanced solid tumors or multiple myeloma and brain cancer (NCT01723020; NCT03107780). B1-907828, another potent mdm2 inhibitor, suppressed cell viability (IC50 of 21.1–424.9 pM) on wt-p53 human glioma tumor stem cells (GTSC) with several Mdm2 amplification levels and different MGMT promoter methylation statuses. It also induced apoptosis, p21 and Puma expression, and PARP cleavage via p53 in some wt-p53 GTSC, but not in mutant (mut-) p53 GTSC. Furthermore, administering B1-907828, either as a single agent or in combination with TMZ, significantly increased the survival of mice with intracranial GTSC-derived tumors. The authors suggested that B1-907828 can cross the BBB [328]. 

#### 9.1.2. Dual mdm2/mdmx Inhibitors

mdm2/mdm4 antagonists have been developed to disrupt the p53/mdm2/mdm4 complex and reactivate the p53 pathway in wt-p53 cancer cells (Table 1). Pazgier et al. reported that a peptide inhibitor (TSFAEYWNLLSP) named PMI bound mdm4 and mdm2 with a Kd of 9 and 3nM, respectively [329]. Xiang et al. reported that the PMI analogous PMI-1 (TSFAEYWNLLfP) and PMI-1-4 (TSFAEYWNLLyP) failed to decrease U87 cell viability even at high concentrations (0–30 μM); the authors suggested that neither compound could cross the plasma membrane [330]. Interestingly, liposome encapsulation increased the antiproliferative activity of both peptides on U87 cells, with an IC50 of 26.3 ± 0.3, 2.24 ± 0.1, and 8.71 ± 0.1 μM for PMI liposomes, PMI-1 liposomes, and PMI 4-liposomes, respectively. PMI-1 liposomes induced cell cycle arrest and apoptosis on wt-p53 U87 cells but had no effect on U251 glioma cells with mut-p53, demonstrating a dependence on wt-p53 [330]. Another mdm2/mdm4 inhibitor, the RGD-M/sPMI peptide formulated in micelles, inhibited proliferation in vitro and induced cell cycle arrest and apoptosis in U87 glioma cells via a p53-dependent mechanism involving the transcription of its target genes p21 and mdm2; it also reduced tumor volume and weight in subcutaneous tumor-bearing mice. The peptide RGD-M/sPMI also increased median survival in intracranial tumor-bearing mice. A combination of RGD-M/sPMI and TMZ showed higher therapeutic efficacy in both models [331].

#### 9.1.3. Restoring p53 Function

Most TP53 missense mutations with a role in GBM are located at the DNA-binding domain, suppressing its transcriptional capacity (Table 1). Thus, mut-p53 with oncogenic functions is overexpressed as it cannot bind to mdm2. Therefore, reactivating the function of wt-p53 could be an effective therapy for GBM. The small molecule p53-reactivation and induction of massive apoptosis-1 (PRIMA-1) can restore mut-p53 native folding and transcriptional capacity by alkylating its thiol groups [332]. PRIMA-1 significantly suppresses proliferation in mut-p53 glioma cells by inducing the transcription of genes positively regulated by p53, such as p21cip1, the growth arrest and DNA damage gene 45a, and mdm2. Thamires Magalhaes et al. reported that PRIMA-1 potentiates sensitization to ionizing radiation-induced by Rho GTPase inhibition in mut-p53 U138MG glioma cells by reactivating p53 and reducing the activity of DNA repair pathways [333]. A combination of doxorubicin and PRIMA-1 had a synergistic effect on p53 accumulation in glioma cells, increasing p53 refolding, inducing p21 and bax, downregulating Bcl-2, and promoting apoptosis [334]. PRIMA-1MET, a methylated analog of PRIMA-1, is more potent and has better permeability than PRIMA-1 [335,336]. Patyka et al. reported that PRIMA-1MET had an anti-proliferative effect on human GSCs, disrupting the structure of neurospheres independently of p53 status. The authors demonstrated that PRIMA-1MET promotes wt-p53 activation, decreasing MGMT levels in MGMT- and p53-positive GSCs or decreasing mut-p53 levels in MGMT- and mut-p53-negative GSCs [337]. The authors suggested that MGMT inactivation increases the sensitivity of GBM cells to PRIMA-1MET [337]. It has been proven that p53 inhibits MGMT expression by inhibiting the transcriptional factor specificity protein 1 (SP1) [338]. Co-treatment with PRIMA-1MET and panobinostat (a HDAC inhibitor), 3-deazaneplanocin (an EZH2 histone methyl transferase inhibitor), or TMZ act synergistically, suppressing the capacity for colony formation on plate and agar, promoting apoptosis in mut- or wt-p53 GBM cells [339]. PRIMA-1MET decreased tumor volume and growth in mice bearing mut-p53 glioma cells in the flank, correlating with low levels of xCT; the authors suggested that PRIMA-1MET exerts its cytotoxic effect by damaging the antioxidant system of GSCs [340]. xCT exports glutamate and imports cystine; on the one hand, increased glutamate levels induce death in brain-surrounding cells; on the other, cystine is a precursor of GSH, which can protect tumor cells from the respiratory burst induced by various therapies [340]. Diverse clinical trials have been evaluated with PRIMA-1MET in phase I/II for the treatment of TP53 mutant myeloid neoplasms and esophageal carcinoma (NCT03072043; NCT02999893; NCT03588078)

Another compound that restores p53 transcriptional activity in mut-p53 glioma cells is P53R3, which has demonstrated superior cancer cell specificity and the ability to target a wider range of p53 mutants than PRIMA-1 [341] (Table 1). Weinmann et al. demonstrated the anti-proliferative effect of P53R3 on mut-p53 LN-308 sublines via cell cycle arrest; in mut-p53 T98G glioma cells, P53R3 induces the expression of genes positively modulated by p53, including mdm2, p21, growth arrest and DNA damage 45 (GADD45), Bax, Fas death ligand (CD95L), Puma, p53-inducible gene 3,-6 (PIG3-6), and death receptor (DR5). It has shown a synergistic effect with tumor necrosis factor-related apoptosis-inducing ligand (TRAIL) in the T98G line, inducing caspase-8 and -3 activation and PARP cleavage [341]. When linked to the carboxyl terminus of p53, the 21-amino acid peptide antennapedia (p53p-Ant) inhibits cell proliferation in correlation with p53 expression levels in human (mut-p53 U138 and wt-p53 U87MG) and rat (F98 and D74, both with mut-p53) glioma cells [342]. p53p-Ant induces extrinsic apoptosis via the Fas/Fas-associated death domain (FADD) pathway by activating caspase-8 and -3 but not caspase-9. It also induces apoptosis in an orthotopic model of 9L glioma [342].

### 9.2. HIF1α Inhibitor Drugs

#### 9.2.1. PX-478

HIF1α is overexpressed in high-grade glioma tissues and is significantly associated with poor survival. Recently, it has been described that HIF1α inhibitors in combination with immune checkpoint inhibitors (ICIs) could enhance the antitumoral immunity in GBM (Table 1). The relationship between PD-L1 and HIF1α in glioma could explain the influence of hypoxia on tumor immune escape. Recently, Ding et al. showed the direct binding of HIF1α to the PD-L1 proximal promoter region, providing evidence that HIF1α upregulates PD-L1 in glioma. In a murine glioma model, they demonstrated the antitumor effect of the HIF1α inhibitor PX-478 when combined with the anti-PD-L1 antibody. This combination reduces tumor growth and increases survival by activating dendritic cells (DC) and CD8 T cells and decreasing PDL-1 expression, which reduces GBM’s immunosuppressive microenvironment [343]. This finding is supported by another study that describes the synergistic effect of PX-478 with anti-PDL-1 to impair tumor growth in vitro and in vivo in non-small cell lung cancer [344]. PX-478 (S-2-amino-3-[4′-N,N,-bis(chloroethyl)amino]phenyl propionic acid N-oxide dihydrochloride) is an orally bioavailable agent that suppresses constitutive and hypoxia-induced HIF1α in cancer cell lines at multiple levels by selectively inhibiting HIF1α translation, reducing its mRNA levels, and inhibiting its deubiquitination [345]. PX-478 has shown antitumor activity against various tumor xenografts by inhibiting HIF1α and its target genes, such as VEGF and GLUT1, inducing glycolysis inhibition [346,347]. In U87 and U259 glioma cells, the inhibition of HIF1α by PX-478 reduced the levels of procollagen-lysin 2-oxoglutarate 5-dioxygenase 2 (PLOD2), a hypoxia-induced enzyme that promotes invasion and migration in GB cells [348]. PX-478 showed an antitumoral effect, either alone or combined with agents such as arsenic trioxide and gemcitabine, in mouse xenograft models, reducing tumor growth [349,350,351]. In esophageal squamous cell cancer (ESCC), PX-478 promotes apoptosis, induces cell cycle arrest in the G2/M phase, represses the epithelial–mesenchymal transition (EMT), and reduces cyclooxygenase-2 (COX-2) and PD-L1 expression in ESCC cells [352]; it also enhances radiosensitivity in prostate carcinoma cells under both normoxic and hypoxic conditions, arrests the cell cycle in S/G2M, and phosphorylates the histone H2AX, inducing double-strand breaks (DSB) in DNA [353]. PX-478 has been evaluated in phase I clinical trials in lymphoma and advanced solid tumors (NCT00522652), and it is a potential drug against hypoxic cells.

#### 9.2.2. Apigenin

Apigenin sensitized glioma to radiotherapy by inhibiting glycolytic enzymes and the expression of NF-κB, p65, HIF1α, GLUT-1/3, and pyruvate kinase isozyme type M2 (PKM2) in human GSCs and a subcutaneous glioma model (Table 1). This treatment reduced cell stemness and DNA damage repair [354,355]. Apigenin (4’,5, 7-trihydroxyflavone) is a natural flavonoid widely distributed in botanical species, including vegetables and fruits such as onions, tea, chamomile, oranges, parsley, and wheat sprouts [356]. It has low toxicity, is nonmutagenic, and has anxiolytic, antioxidant, anti-inflammatory, antiviral, antibacterial, and antitumoral effects. Its anti-inflammatory effects have been linked to signaling pathways such as NF-κB, MAPK/ERK, and c-Jun N-terminal kinases (JNK). Apigenin has shown a broad anticancer effect in various malignancies by inducing autophagy and apoptosis, arresting the cell cycle, inhibiting cell migration and invasion, and stimulating the immune response [243,357,358,359]. Apigenin induced cell death and apoptosis in lung epithelium cancer (A549) [360] and showed a synergistic effect with curcumin [361]. In human ovarian cancer cells, it inhibits the expression of VEGF and HIF1 through the pI3K/AKT/p70S6K1 and homologue of mdm2 (HDM2)/p53 pathways, inducing a mitochondrial death cascade. VEGF expression through HIF1 in response to oncogene activation and growth factor stimulation is activated by PI3K/AKT signaling [362,363,364].

#### 9.2.3. Propofol

Propofol downregulates HIF1α expression in GB and enhances the sensitivity of GB cells to TMZ, reducing tumor growth, macrophage infiltration, and inflammation in TMZ-resistant GBM xenograft tumors (Table 1). Additionally, it reduced the expression of HIF1α, MGMT, p65, and Cox2. By inhibiting macrophage activation, it contributes to reducing immunosuppression [365]. Propofol (2,6-diisopropylphenol) is an intravenous anesthetic agent commonly used to induce and maintain anesthesia. In addition to its effect on reducing pain, propofol has potential antitumor activity. Various reports have shown that propofol induces cytotoxicity in different cancer cells, including glioma [366,367,368]. Shu-Shong et al. reported that it generates cytotoxicity in 8401 human GB cells through ROS-associated apoptosis in a concentration-dependent manner that induces cell cycle G2/M arrest and caspase activation [369]. Propofol inhibited glioma cell proliferation, migration, and invasion by regulating the miR-410-3p/transforming growth factor-B receptor type 2 (TGFBR2) axis, the miR-206/Rho-associated protein kinase 1 (ROCK1) axis, and miR-134 expression, as well as suppressing the PI3K/Akt signaling pathway [370,371,372]. miRNAs have been described to participate in various biological processes under physiological and pathological conditions [373]. MiR-206 acts as a tumor suppressor in several types of cancer, inhibiting proliferation and promoting apoptosis in early-stage glioma, and its decreased expression is associated with poor prognosis [374]. Propofol reduces Glu uptake, lactate production, extracellular acidification, and the expression of HX2, PKM2, LDHA, and GLUT1 in lung cancer cells, affecting aerobic glycolysis through the circRNA transcriptional adaptor 2A (circTADA2A)/miR-455-3p/ fork-head box M1 (FOXM1) axis, suppressing carcinogenesis [375]. Multiple clinical trials have been conducted with propofol, as it is commonly used as an anesthetic. One focuses on determining its impact on brain cancer survival (NCT04962672), while another evaluates its effectiveness as chemotherapy in combination with PDL-1 antibodies (NCT05273827).

#### 9.2.4. Fenofibrate

Fenofibrate modulates multiple cellular pathways that regulate HIF1α and pH-regulating protein CA9; this regulation is mediated by the AMPK/heme oxygenase-1 (HO-1)/SIRT1 pathway in GBM cells [376] (Table 1). Fenofibrate is a peroxisome proliferator-activated receptor α (PPARα) agonist approved by the Food and Drug Administration (FDA) for the treatment of hyperlipidemia. PPAR agonists regulate AMPK, which is a cellular energy sensor as well as a regulator of the metabolic process [377]; AMPK contributes to malignancy by regulating pathways such as HIF, mTOR, and p53 [378]. Anticancer effects have been reported for fenofibrate in various cancer types, including colon, cervical, breast, and prostate, modulating multiple signaling pathways to induce apoptosis, cell cycle arrest, and suppress cell proliferation [379,380,381,382]. Fenofibrate inhibited cell invasion and migration in oral squamous cell carcinoma (OSCC) by blocking NF-kB signaling [383]. This compound has been evaluated in patients with smoldering or symptomatic multiple myeloma (NCT01965834); it was also assessed in a phase II trial along with etoposide, cyclophosphamide, thalidomide, and celecoxib in children with recurrent or progressive cancer (NCT00357500), and in patients with medulloblastoma, ependymoma, and atypical teratoid rhabdoid tumor (ATRT) using bevacizumab in combination with other five oral drugs, including fenofibrate (NCT01356290).

#### 9.2.5. Resveratrol

Resveratrol (3,5,40-trihydroxy-trans-stilbene) is a natural polyphenolic compound in peanuts, grapes, and other fruits [384]. Various studies have reported its antineoplastic properties, showing protective and antiproliferative effects against several types of cancer [385,386] (Table 1). This compound exerts its action by modulating various signaling pathways and molecular targets in cancer, such as Notch, Janus kinase (JAK)/STAT, and NF-κB [387,388]. It is an inhibitor of HIF1α, regulating its translation and degradation [388,389,390,391]. It induces cell cycle arrest in the G0/G1 phase, suppressing cyclin D1 expression and inhibiting cell proliferation in prostate cancer-derived cell lines [392]. Resveratrol enhances TMZ cytotoxicity in GB cells [393,394]. Combined with iododeoxyuridine (IUdR), resveratrol enhanced the radiosensitization of GBM spheroid cells, reducing colony counts and increasing DNA damage on U87MG glioblastoma cells; this treatment could improve the therapeutic index for radiation [395]. In ovarian cancer, resveratrol reduces tumor growth, inhibiting proliferation, migration, invasion, and apoptosis induction; these effects are mediated by the inhibition of glycolysis and activation of the AMPK/mTOR signaling pathway [396]. Resveratrol also modulates lipid metabolism in cancer, reducing lipid synthesis by inhibiting SREBPs, which are a target of AMPK; in cancer patients, a reduction in metastasis and tumor volume has been associated with decreasing serum TAG, VLDL, and LDL levels [397,398,399]. Resveratrol modulates the activity and expression of sirtuins, NAD-dependent enzymes involved in cellular mechanisms such as Glu and lipid metabolism, DNA repair, and tumorigenesis [397,400]. Additionally, resveratrol can cross the BBB, which is an advantage when used against CNS tumors [401]. Resveratrol has been evaluated in clinical trials as a chemopreventive agent in healthy subjects (NCT00098969) and a chemotherapeutic agent in colon cancer and neuroendocrine tumors (NCT00256334, NCT00433576, and NCT01476592).

### 9.3. c-Myc Inhibitor Drugs

#### 9.3.1. Inhibitors of c-Myc Transcriptional Activity

Verteporfin, a porphyrin derivative, has been reported to suppress cell proliferation in SNB19 and LN229 glioma cells in vitro by decreasing the expression of c-Myc, octamer-binding transcription factor 4 (OCT4), the connective tissue growth factor (CTGF), VEGFA, and survivin, molecules involved in cell growth, angiogenesis, migration, invasion, apoptosis resistance, and tumor recurrence in glioma; these results correlated with a decrease in the Yes-associated protein (YAP)/transcriptional co-activator with PDZ-binding motif (TEAD) signaling and activation of p38, a pro-apoptotic MAPK [402]. Vigneswaran et al. reported that the YAP/TAZ-TEAD pathway directly regulated c-Myc, EGFR, and SOX-2 transcription in human GSC with mutant EGFR. The inhibition of YAP/TAZ-TEAD transcriptional activity by verteporfin induced a pro-apoptotic effect in GSC by binding to YAP and TAZ, promoting the degradation of both transcriptional coactivators, preventing their nuclear localization, and decreasing the expression of their transcriptional targets. The authors suggested that verteporfin induces the transcription of c-Myc via EGFR/ large tumor suppressor 1 and 2 (Lats 1/2)/YAP/TAZ/TEAD [403]. Furthermore, a study reported that verteporfin is absorbed and accumulated in tumor cells of human GBM, supporting its therapeutic potential for GBM with mutant or amplified EGFR [403] (Table 1). Diverse clinical trials in phases I/II have been evaluated with verteporfin; these are for recurrent glioblastoma, recurrent prostate cancer, breast neoplasms, and advanced pancreatic carcinoma (NCT04590664; NCT03067051; NCT02872064; NCT03033225).

Other drugs that inhibit c-Myc transcription in glioma cells are CUDC-907 and JQ-1. JQ-1, a pan-inhibitor of bromodomain and extra-terminal (BET) proteins such as the epigenetic readers BRD2, BRD3, and BRD4, exerts a cytotoxic effect on BT142 glioma cells with mutated IDH, by inhibiting BRD4 and c-Myc downregulation [404]. Delmore et al. showed that BRD4 inhibition by JQ-1 decreases c-Myc transcription, repressing genes induced by the oncogene c-Myc [405]. Wen et al. reported that JQ-1 and siBRD4 show an antineoplastic effect on GSCs in vitro and in vivo by inhibiting the VEGFR/PI3K/AKT signaling pathway. The authors suggested that AKT inactivation induced cell cycle arrest by upregulating the CDK inhibitors p21 and p27, inhibiting the CDK/Cyc D/ E2F pathway, and promoting c-Myc downregulation; AKT inactivation also induces apoptosis by upregulating pro-apoptotic proteins such as Bax, Bim, Bak, and caspase-3 [406] (Table 1).

A combination of CUDC-907 (a dual HDAC/PI3K inhibitor) and radiotherapy has shown synergistic antitumor activity on c-Myc-amplified BT245 and SF188 glioma cell lines, downregulating NF-κB and FOXM1 expression and transcriptional activity, blocking DNA repair, enhancing radiosensitization in glioma cells, and activating apoptosis [407]. CUDC-907 was also suggested to modulate c-Myc expression in glioma cells [407]. Sun et al. demonstrated that CUDC-907 suppressed c-Myc expression, inducing apoptotic cell death in c-Myc-dependent cancer lines [408] (Table 1). Diverse clinical trials in phases I/II have been evaluated with CUDC-907; these are for thyroid neoplasms, breast and ovarian cancer, lymphoma, neuroblastoma, recurrent glioblastoma, and prostate cancer (NCT03002623; NCT02307240; NCT02909777; NCT03893487; NCT02913131).

HDAC inhibitors such as panobinostat (Pb), vorinostat (Vr), and romidepsin (Ro) have been used in glioma models targeting the Warburg effect, disrupting super-enhancers like c-Myc itself. c-Myc suppression leads to upregulation of the transcription factors peroxisome proliferator-activated receptor γ coactivator 1 α (PGC1α) and peroxisome proliferator-activated receptor δ (PPARδ), both activators of oxidative metabolism. This approach could be useful for treating glioma [308].

A selective HDAC8 inhibitor named NBM-BMX (BMX) has been used to enhance the effect of TMZ on treatment-resistant glioma cell lines. BMX seems to overcome TMZ resistance by downregulating the β-catenin/c-Myc/SOX2 signaling pathway and upregulating p53, which in turn mediates the inhibition of MGMT, a DNA repair factor [409] (Table 1). Furthermore, this compound has been evaluated in phase I/II studies in patients with malignant neoplasm or combined with the standard chemotherapy in newly diagnosed glioblastoma (NCT06012695; NCT03726294; NCT03808870).

Epigenetic regulation by demethylase inhibitors is a promising approach. KDM4C is a histone H3K9 demethylase overexpressed in glioma cells, which binds to the c-Myc promoter, inducing its expression and decreasing apoptosis. Pharmacological inhibition to treat glioma is currently an active field of research [302]. 

Anthracenyl-isoxazole amides have shown antitumor activity. Recently, 10-anthryl-oxy-isoxazole-pyrrole-doubletails (RO-AIMs) have shown promise as antitumor drugs. Based on in silico analysis, it has been postulated that RO-AIMs exert their effect by interacting with the DEAH-Box helicase 36 (DHX36) and c-Myc in a ternary complex, halting glioma cell proliferation [410] (Table 1).

Telomestatin is another intercalating agent isolated from *Streptomyces anulatus*. A telomestatin derivative named S2T1-6OTD binds to the G-quadruplex structure in the c-Myc promoter, inhibiting its expression and activity; growth arrest and apoptosis induction in brain cancer cell lines were observed, including glioma [411] (Table 1).

#### 9.3.2. Inhibition of the c-Myc-Max Heterodimer

KJ-Pyr-9 suppressed cell viability in c-Myc-amplified CSCs by blocking the c-Myc-Max heterodimerization in coadministration with TMZ [412] (Table 1).

In other strategies, the c-Myc inhibitory peptide (H1) has been proposed, coupling it to carriers such as elastin-like polypeptide (ELP) and cell-penetrating peptides to enhance its delivery to brain tumor cells. H1, derived from the helical loop domain of c-Myc, blocks the interaction between c-Myc and Max, preventing the transcription of both c-Myc and Max. This approach was evaluated in vivo with promising results in reduced tumor volume and increased survival time of animal subjects, demonstrating that the specificity of target delivery can improve treatment efficacy [413] (Table 1).

MYCi975, a small molecule, binds directly to the HLH domain of c-Myc, decreasing its transcriptional activity [414], blocking the formation of the mediator subunit 1 (MAD)/c-Myc complex, and inhibiting the transcription of TMEM44-AS1, an lncRNA, thereby reducing cell viability and the ability to form colonies in glioma cells. TMEM44-AS1 induces proliferation, migration, and invasion in LN-18 and U251 glioma cells via the TMEM44-AS1/Serpin B3/c-Myc and TMEM44-AS1/Serpin B3/EGR1/IL-6 signaling pathways [415]. In vivo, MYCi975 promotes c-Myc degradation by blocking c-Myc/Max formation, reducing tumor volume, and increasing immune cell infiltration at the tumor site [416] (Table 1).

**Table 1:** Inhibitors of p53, HIF1, and c-Myc in glioma.

Target	Treatment	Preclinical and Clinical Trials (Clinical Trial Number)	References
Inhibitor of mdm2	Nutlin3	Primary cultures from patients with glioblastoma treated with 10 mM Nutlin3 showed decreased cell proliferation and increased apoptosis. It also induced changes in Puma, Noxa, and Survivin gene expression in wild-type p53 samples.	[318]
Inhibitor of mdm2	RG7112 analogue of nutlin	In the orthotopic GBM model (3731) amplified with mdm2, RG7112 decreased the rate of tumor progression and prolonged the survival of mice compared to vehicle-treated mice.	[321]
Inhibitor of mdm2	RG7388 analogue of nutlin	In U87 glioblastoma cells, short-term treatment with RG7388 (100 nmol/L) for 72 h caused an increase in cells in the G^1^ cell cycle phase compared to the control group. (Clinical trial number: NCT03158389)	[323]
Inhibitor of mdm2	AMG232	A study was carried out in 10 patients with glioblastoma, and AMG232 (240 mg) was administered, presenting a significant increase in serum MIC-1 with respect to the initial value 24 h after the single dose in all 10 patients. It Is suggested that high serum MIC-1 values are an indicator of tumor suppression. (NCT01723020; NCT03107780)	[325]
Inhibitor of mdm2	B1-907828	BI-907828 decreases viability increases cell death, and increases transcriptional regulation of p53, p21, and Puma through p53-dependent cytostasis and apoptosis.	[325]
Dual mdm2/mdmx inhibitors	TSFAEYWNLLSP	Potent dual L peptide inhibitor PMI (TSFAEYWNLLSP) of p53-mdm2/mdmx interactions, encapsulated in a liposome, has a significant antitumor effect by inducing apoptosis and decreasing the synthesis phase of the cell cycle in U87 cancer cells.	[330]
Dual mdm2/mdmx inhibitors	TSFAEYWNLLSP/ RGD- M/sPMI/ TMZ	The combination of the two compounds generated arrest of the cell cycle, increased apoptosis, decreased the volume and weight of the tumor and prolonged the survival of mice.	[331]
Restoration of p53	PRIMA	PRIMA-1^MET^ induces cytotoxic effects in GBM cell lines independently of p53 status.	[337]
Restoration of p53	PRIMA	Treatment with PRIMA-1^Met^ significantly reduces tumor volume and decreases the p53 protein and the cystine-glutamate antiporter system, which regulates the homeostasis of excitatory neurotransmitters in the brain. (NCT03072043; NCT02999893; NCT0358807)	[340]
Restoration of p53	P53R3	P53R3 induces cell cycle arrest at specific stages (S phase and a G0/G1 cell cycle arrest) and generally slows cell cycle progression, depending on the p53 mutant. P53R3 and APO2L.0 can activate caspase 3 to generate death by apoptosis.	[341]
Inhibitor to HIF1α	PX-478	PX-478, combined with anti-PD-L1 antibody, decreases tumor growth, and increases survival by activating dendritic and CD8 T cells. PX-478 marked antitumor activity in large tumor xenografts, accompanied by massive apoptosis. (NCT00522652)	[322,346]
Inhibitor to HIF1α	Apigenin	Apigenin (80µM) inhibits cell viability in SW480 and HCT15 colorectal cancer cells. Apigenin (100µM) Inhibits Human Cervical Cancer Cell Viability and Induces Cell Cycle Arrest (sub G1 phase).	[357,359]
Regulates HIF1α expression	Propofol	Propofol enhances the sensitivity of TMZ-resistant GBM in vivo, decreases tumor growth, and enhances the effect of TMZ on macrophage infiltration, inflammation, and apoptosis. Propofol (5 or 10 μg/mL) activated miR-410-3p expression and inhibited TGFBR2 expression in glioma cells, generating inhibition of glioma cell development. (NCT04962672; NCT05273827)	[365,370]
Regulates HIF1α expression	Fenofibrate	Fenofibrate administration inhibited colon cancer cell proliferation and significantly decreased tumor volume in tumor xenograft experiments using HCT116 cells, weakening DNMT1 activity and restoring CDKN2A expression. Fenofibrate had anticancer effects on cervical cancer HeLa cells via inducing caspase-dependent apoptosis and cell cycle arrest. (NCT01965834;NCT00357500;NCT01356290)	[379,382]
Regulates the HIF1α synthesis and degradation	Resveratrol	Resveratrol modulates signaling pathways such as Notch, JAK/STAT, and NF-κB in cancer cells. Resveratrol increases the TMZ cytotoxicity in glioma cells. Resveratrol plus iododeoxyuridine enhanced the radiosensitization of U87MG spheroids. (NCT00098969; NCT00433576; NCT01476592)	[387,388,393,394,395]
Inhibitor of c-Myc transcriptional activity	Verteporfin	Inhibits the cell viability in glioma cells via YAP/TEAD signaling pathway inhibition. YAP/TAZ-TEAD signaling regulates the c-Myc transcription. Verteporfin is absorbed and accumulated in human GBM tumors. (NCT04590664; NCT03067051; NCT02872064; NCT03033225)	[402,403]
Inhibitor of c-Myc transcriptional activity	JQ-1	JQ-1 suppresses the c-Myc transcription of BT142 cells (IDH mutated) via inhibition of BRD4. JQ-1 plus siBRD4 induces cell cycle arrest and apoptosis in glioma cells through down-regulation of VEGFR/PI3K/AKT signaling.	[404,405,406]
Inhibitor of c-Myc transcriptional activity	CUDC-907	CUDC-907 plus radiotherapy increases the radio sensitization in glioma cells with c-Myc amplified, promoting apoptosis. (NCT03002623; NCT02307240; NCT02909777; NCT03893487; NCT02913131).	[341]
Inhibitor of c-Myc transcriptional activity	NBM-BMX (BMX)	NBM-BMX (BMX) enhancer the sensitiveness at TMZ in glioma cells by inhibiting the activity of the β-catenin/c-Myc/SOX2 signaling pathway. (NCT06012695; NCT03726294; NCT03808870)	[409]
Inhibitor of c-Myc transcriptional activity	RO-AIMs	Suppress the cell proliferation in glioma cells by forming RO-AIMs/ helicase DHX36/ c-Myc complex that inhibits c-Myc.	[410]
Inhibitor of c-Myc transcriptional activity	S2T1-6OTD	Induces apoptosis via downregulation of c-Myc transcription in glioma cells.	[411]
Complex c-Myc/Max inhibitor	KJ-Pyr-9	KJ-Pyr-9 plus TMZ inhibits cell proliferation in glioma cells by blocking the c-Myc-Max heterodimerization.	[412]
Complex c-Myc/Max inhibitor	c-Myc inhibitory peptide (H1)	Treatment of H1 on Rat Glioma Model reduces tumor volume and increases the survival of rats by inhibiting at c-Myc.	[413]
Complex c-Myc/Max inhibitor	MYCi975	MYCi975 inhibits cell proliferation, migration, and invasion by reducing c-Myc/TMEM44 AS1 signaling.	[415]

## 10. Conclusions

Studies carried out by many researchers conclude that the metabolic reprogramming in glioblastoma results from genetic alterations, in which the signaling pathways RAS/RAF/MERK/ERK and PI3K/AKT/mTOR regulate glucose, nucleotide, protein, and lipid metabolism to support glioma cell proliferation, growth, migration, and invasion. These signaling pathways are crucial for glioma cells to respond and adapt to external stressors by altering their mechanisms of response at both transcriptional and translational levels, modulating p53, HIF1, and c-Myc to promote glycolysis first and catabolism later. It should be noted that p53 usually shows opposite effects to HIF1 and c-Myc. p53 inhibits glycolysis, the pentose phosphate pathway, and lipid and nucleotide synthesis, activating oxidative phosphorylation and fatty acid oxidation. Meanwhile, HIF1 and c-Myc induce the transcription of most enzymes that participate in glycolysis, the pentose phosphate pathway, glutaminolysis, and lipid, cholesterol, and nucleotide synthesis, inhibiting electron transport chain complexes and enzymes of the tricarboxylic acid cycle and β-oxidation. p53 could activate signaling pathways that promote survival under several conditions, including transient hypoxia, or act synergistically with HIF1 under more severe or more prolonged hypoxia. c-Myc can induce metabolic stress, promoting apoptosis via AMPK/p53 [417]. Moreover, the activation of p53 and c-Myc could modulate various metabolic activities induced by HIF1, and vice versa. However, integrating and deepening our knowledge of the cellular and molecular processes induced by p53, HIF1, and c-Myc, as well as the crosstalk between them and the signaling pathway that regulates their crosstalk with components of the microenvironmental including cells of the immune system, are essential for understanding glioblastoma and devising novel therapeutic approaches. Above all, therapeutic interventions overcome the blood–brain barrier and that could modulate the immune cell infiltration and tumor microenvironment. Drugs target the RAS/RAF/MERK/ERK and PI3K/AKT/mTOR signaling pathways combined with p53, HIF1, and c-Myc inhibitors. Collective studies are also required to determine the resistance mechanisms that can occur during metabolic reprogramming and how they can be prevented. Unfortunately, glioblastoma is an extremely inter- and intra-heterogeneous tumor with high plasticity [418], so it is an extremely complicated task. Despite this, many positive results allow us to think that the fight against tumor metabolism is and will be an efficient fight against cancer.

## Data Availability

Not applicable.
